# Electro-osmotic Drag and Thermodynamic Properties of Water in Hydrated Nafion Membranes from Molecular Dynamics

**DOI:** 10.1021/acs.jpcc.2c01226

**Published:** 2022-05-03

**Authors:** Ahmadreza Rahbari, Remco Hartkamp, Othonas A. Moultos, Albert Bos, Leo J. P. van den Broeke, Mahinder Ramdin, David Dubbeldam, Alexey V. Lyulin, Thijs J. H. Vlugt

**Affiliations:** † Engineering Thermodynamics, Process & Energy Department, Faculty of Mechanical, Maritime and Materials Engineering, Delft University of Technology, Leeghwaterstraat 39, 2628CB Delft, The Netherlands; ‡ XINTC global, Wesselseweg 134, 3774 RL Kootwijkerbroek, The Netherlands; ¶ Van’t Hoff Institute for Molecular Sciences, University of Amsterdam, Science Park 904, 1098XH Amsterdam, The Netherlands; § Soft Matter and Biological Physics, Department of Applied Physics, Eindhoven University of Technology, P.O. Box 513, 5600 MB Eindhoven, The Netherlands; ∥ Center for Computational Energy Research, P.O. Box 6336, 5600 HH Eindhoven, The Netherlands

## Abstract

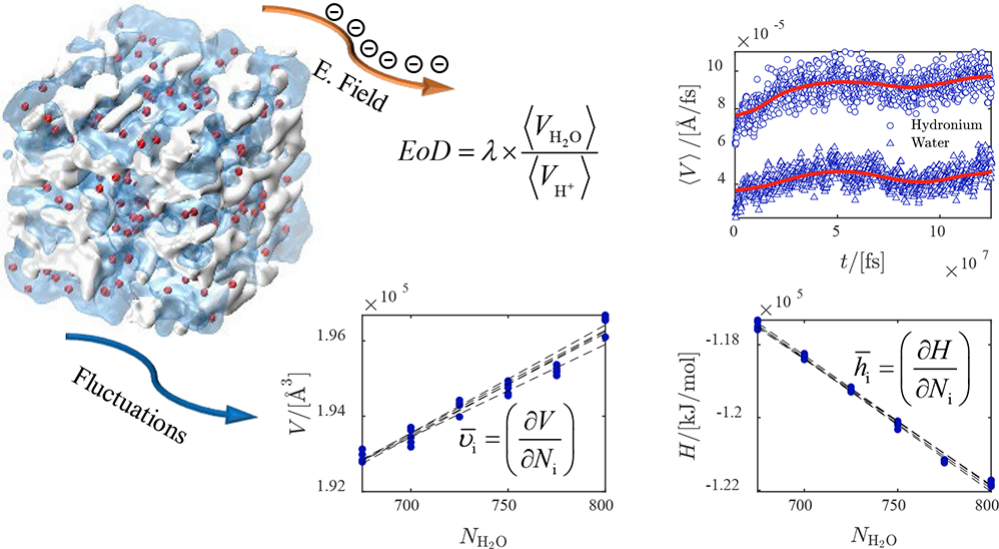

One of the important parameters in water management of proton exchange membranes is the electro-osmotic drag (EOD) coefficient of water. The value of the EOD coefficient is difficult to justify, and available literature data on this for Nafion membranes show scattering from in experiments and simulations. Here, we use a classical all-atom model to compute the EOD coefficient and thermodynamic properties of water from molecular dynamics simulations for temperatures between 330 and 420 K, and for different water contents between λ = 5 and λ = 20. λ is the ratio between the moles of water molecules to the moles of sulfonic acid sites. This classical model does not capture the Grotthuss mechanism; however, it is shown that it can predict the EOD coefficient within the range of experimental values for λ = 5 where the vehicular mechanism dominates proton transfer. For λ > 5, the Grotthuss mechanism becomes dominant. To obtain the EOD coefficient, average velocities of water and ions are computed by imposing different electric fields to the system. Our results show that the velocities of water and hydronium scale linearly with the electric field, resulting in a constant ratio of ca. 0.4 within the error bars. We find that the EOD coefficient of water linearly increases from 2 at λ = 5 to 8 at λ = 20 and the results are not sensitive to temperature. The EOD coefficient at λ = 5 is within the range of experimental values, confirming that the model can capture the vehicular transport of protons well. At λ = 20, due to the absence of proton hopping in the model, the EOD coefficient is overestimated by a factor of 3 compared to experimental values. To analyze the interactions between water and Nafion, the partial molar enthalpies and partial molar volumes of water are computed from molecular dynamics simulations. At different water uptakes, multiple linear regression is used on raw simulation data within a narrow composition range of water inside the Nafion membrane. The partial molar volumes and partial molar excess enthalpies of water asymptotically approach the molar volumes and molar excess enthalpies of pure water for water uptakes above 5. This confirms the model can capture the bulklike behavior of water in the Nafion at high water uptakes.

## 1 Introduction

Perfluorinated sulfonic acid (PFSA) membranes are ion-conductive polymer materials used in polymer physics and electrochemistry as solid electrolytes.^1,2^ Because of their high ionic conductivity, PFSA membranes are used as proton-exchange membranes (PEMs) in fuel cells.^1,3−5^ The physical and transport properties of PFSA membranes are studied as a cross-disciplinary research field between polymer physics and electrochemistry.^1,5^

**Figure 1:**
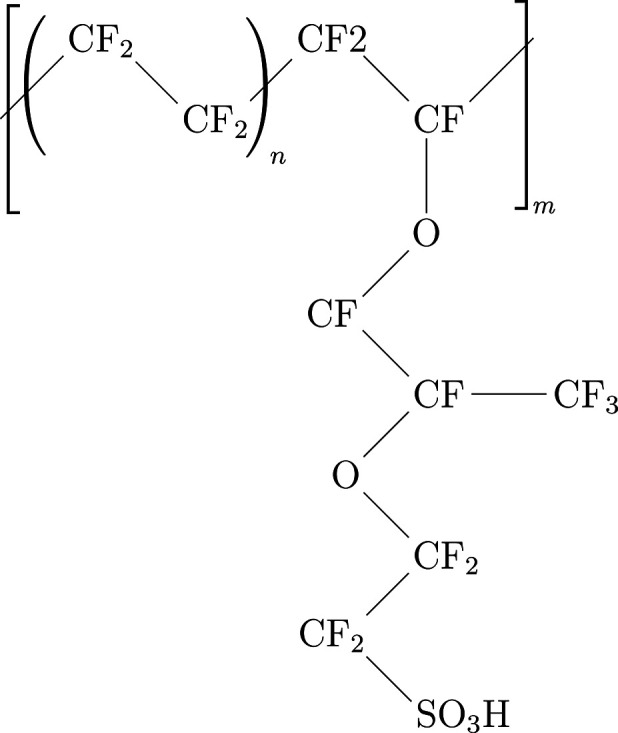
Structure of the Nafion chains used in this study, *n* = 7 and *m* = 10, with the equivalent molecular weight of 1100 g/mol_HSO_3__.^3,12^

A commonly used PFSA membrane is Nafion,^1,5−8^ which is a registered trademark by E. I. DuPont De Nemours & Co.^1,9^ Nafion membranes are chemically inert with a mechanically robust matrix, making them one of the most recognized electrolytes since the 1970s.^1^ A Nafion membrane usually operates in a temperature range below 100 °C.^10,11^ A Nafion monomer has an electrochemically neutral semicrystalline polymer backbone and a side chain with a pendant sulfonic group (HSO_3_).^1,11^ As a PFSA, a Nafion membrane has a hydrophobic polytetrafluoroethylene (PFTE) backbone. The side chains are composed of polysulfonyl fluoride vinyl ether, and sulfonic acid groups are attached to the hydrophilic tail.^3,12^ The polymer backbone is hydrophobic, while the side chain is hydrophilic. In this study, we consider Nafion 117 membranes with an equivalent weight (EW) of 1100 g/mol_HSO_3__, i.e., grams of polymer per equivalent of sulfonate groups.^9^ A chemical representation of the Nafion monomer is shown in Figure 1.

**Figure 2:**
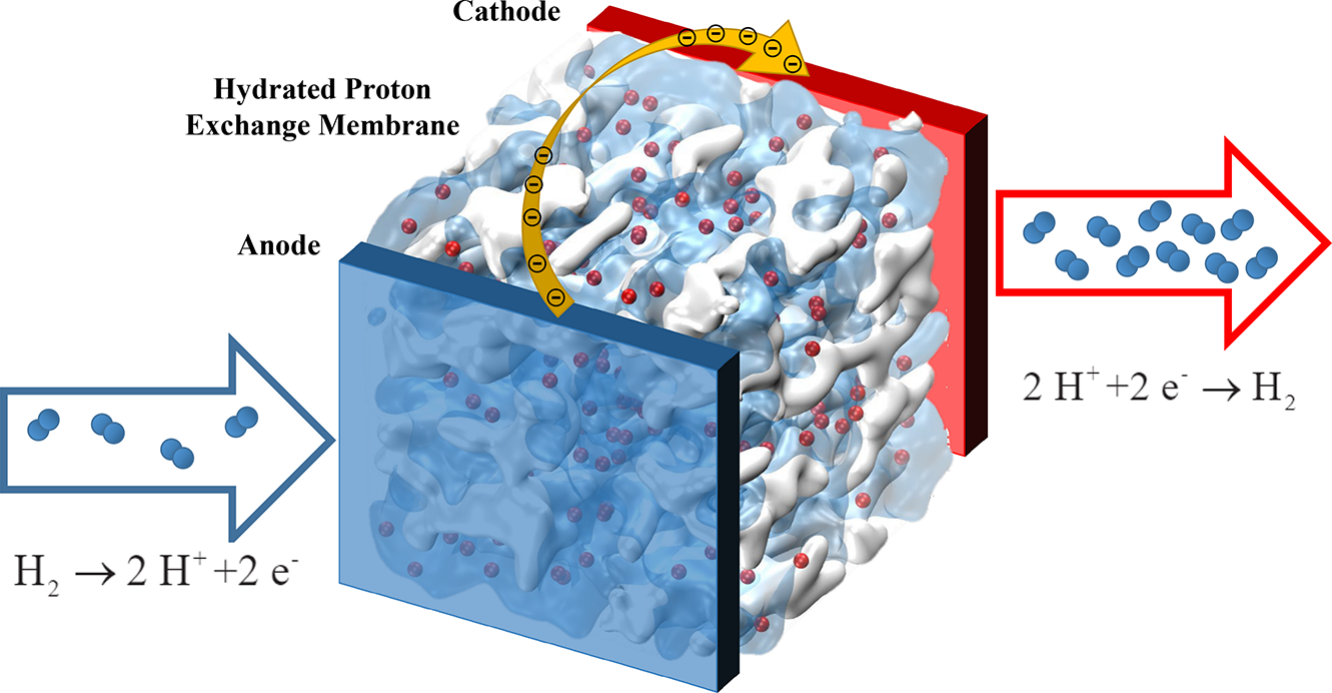
Schematic representation of a membrane inside an electrochemical hydrogen compressor.^81^ At the anode side, hydrogen is split at low pressure and the protons are transferred across the hydrated membrane in the presence of an electric field. At the cathode side, the protons and electrons form hydrogen molecules. Continuing this process leads to increasing pressure at the cathode (high pressure side).

One of the important applications of Nafion membranes is in Electrochemical Hydrogen Compressors (EHCs)^5,11,13,14^ and fuel cells.^1,15^ The membrane inside the EHC facilitates proton transfer and prevents mixing of streams between the anode and the cathode.^12^ The basic working principles of the membrane inside an EHC are the same as those of a PEM inside a fuel cell.^11,15^ The hydrogen at the anode side (low pressure) is split into protons and electrons. The electrons follow an external path while protons are transported through the membrane under the influence of an electric field.^11^ At the cathode side (high pressure), protons are reduced to hydrogen molecules. The working principle of an EHC is schematically shown in Figure 2.

The presence of water in PFSA membranes is crucial for proton conductivity and, therefore, for the performance of the EHC. A low water uptake in the membrane leads to low ionic conductivity, while excessive water can overflow the membrane channels and diminish the performance of the membrane.^4,8^ During hydration, water molecules form hydrogen bonds with the sulfonic acid sites (SO_3_H + H_2_O ↔ SO_3_^–^ + H_3_O^+^), and with the increase in water uptake the dissociated protons from the sulfonic sites will mobilize and form ions such as the hydronium ion H_3_O^+^, Zundel ion, H_5_O_2_^+^, or Eigen ion H_9_O_4_^+^.^1,15^ The water uptake, also known as the water content, λ, is the number of water molecules per sulfonic sites in the Nafion side chain^1,5,16^
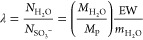
1in which , and  are the number of water molecules and sulfonic acid groups in the system, respectively.  and *M*_P_ the are mass of the absorbed water and dry polymer, respectively. The term EW is defined as the molecular weight of the polymer per sulfonic acid group,^3,12^ and  is the molar mass of water. Physical and thermodynamic properties of the hydrated membrane depend partly on the water uptake in the membrane.

One of the important parameters to be managed for PFSA membranes is the electro-osmotic drag (EOD) coefficient.^17−21^ It is defined as the ratio between the number of water molecules and protons transferred across the membrane in the absence of gradients in concentration and pressure and at vanishing electric field^1−3,18^
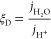
2Here, ξ_D_ is the EOD coefficient of water in the membrane,  is the flux of water, and  is the flux of protons across the membrane in the presence of the electric field. While the definition of the EOD coefficient is straightforward, many attempts have been made to rationalize and quantify this parameter using experiments and simulations.^1,15,19,22−25^ As it will be discussed in this paper, the results of different experiments and simulation methods show a large scattering.

**Figure 3:**
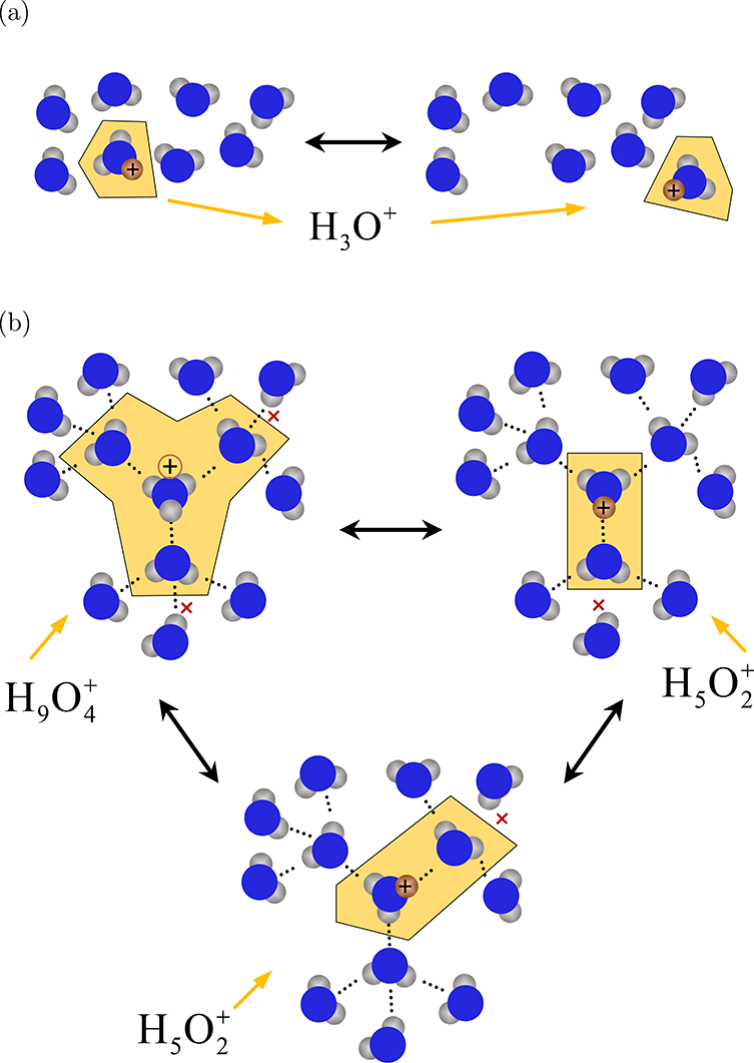
Schemtatic representation of proton transport in water: (a) vehicular mechanism and (b) proton hopping (Grotthuss) mechanism.^27^ The red crosses indicate breaking and formation of hydrogen bonds during proton hopping. H_3_O^+^, H_5_O_2_^+^, and H_9_O_4_^+^ are the hydronium ion, Zundel ion, and Eigen ion, respectively. This schematic representation is based on the work of Jiao and Li.^15^

Polymer membranes have a high proton conductivity which has consequences for the EOD coefficient of water. Free protons do not exist in the mixture due to the lack of a electron cloud.^1^ Protons are transported on water molecules and form dynamic species such as the hydronium ion, H_3_O^+^, Zundel ion, H_5_O_2_^+^, or Eigen ion H_9_O_4_^+^.^1,26^ This form of diffusion for water–proton is known as the vehicular mechanism.^15^ The transport of protons via the vehicular mechanism is schematically shown in Figure 3a. At low water contents, the proton is transferred either directly between the charged sites in the polymer side chain or the vehicular mechanism.^15^ With the increase in the water uptake, the charged sites in the membrane are connected via a continuous network of water agglomerates. In this configuration, the protons can hop from one water molecule to another one. This proton transfer is the so-called hopping mechanism or the Grotthuss mechanism.^1,15,19,27^ The Grotthuss mechanism is schematically shown in Figure 3b. By hopping between water molecules, a proton can form a Zundel ion or an Eigen ion by breaking hydrogen bonds and forming new hydrogen bonds.^1,15,26,28,29^ Since the proton conductivity drops significantly for λ < 5,^15^ this can be considered as an estimate up to which vehicular mechanism dominates the drag of water molecules, however, the exact threshold is unclear.^15^

Different experimental techniques and different conditions are used to report the EOD coefficient in Nafion membranes; however, the results show a large scattering.^1,2^ The experimentally measured EOD coefficients for Nafion membranes and other proton exchange membranes are not always consistent and vary between 0.2 and 9.5, depending on the method used and whether an electrolyte is present. An overview of measurement techniques for EOD coefficients provided by Pivovar^26^ is listed in Table 1. Due to discrepancies between experimental data as shown in refs (1), (2), (25), and (26), it is challenging to reach a general consensus on the value of the EOD coefficient as a function of water content. Other attempts were made to compute the EOD coefficients from molecular simulations. Din and Michaelides^30^ computed the EOD coefficient of water in pores from their model with a linear relation between the EOD coefficient and the water uptake. This significantly overestimates the EOD coefficient at high water contents. It is important to note that the model of Din and Michaelides^30^ is not sufficiently detailed at the molecular level. Choe et al.^19^ computed the EOD coefficient of water from first-principles molecular dynamics of small systems. The number of molecules in a unit cell system size were 19 and 53. The computed EOD coefficients from their study are 0.92 and 1.23 for λ = 4.1 and λ = 12.7. The model of Choe et al.^19^ is complex and it captures the Grotthuss mechanism, however, it uses very small system sizes. For small system sizes, features such as microstructure of the Nafion membrane may not be captured, and the results may be affected by finite size effects.

**Table 1:** Electro-osmotic Drag (EOD) Coefficients of Protons in Nafion Membranes Measured with Different Methods^a^

method	EOD range	source
electro-osmotic drag cell (applied potential)	0.9–4.0	Breslau et al.,^65^ Zawodzinski et al.^66^
radiotracer method (applied potential)	3–8	Mayer and Woermann,^67^ Pivovar et al.,^68^ Verbrugge and Hill^69^
methanol fuel cell (electrochemical)	0.5–5.1	Ren et al.,^70,71^ Pivovar et al.,^72^ Hickner,^73^ Kim et al.^74^
hydrogen pump (applied potential)	0.2–0.6	Weng et al.^75^
electrolysis (applied potential)	0.2–9.5	Balko et al.^76^
		Motupally et al.^77^
streaming potential (applied pressure)	3.5–3.8	Trivijitkasem et al.^78^
		Okada et al.^79^
activity gradient	0.95–1.4	Fuller et al.,^80^ Zawodzinski et al.^58^
electrophoretic NMR (applied potential)	1.5–2.8	Ise et al.^2^

a The EOD coefficient varies roughly from 0 to 5 depending on the method used. For details on the methods, the reader is referred to ref (26).

In the first part of this study, we investigate the transport of water inside Nafion 117 as a function of the water uptake. As discussed in section 2, we use a classical approach in our simulations, which cannot capture the Grotthuss mechanism. However, this model fills the gap between the work of Din and Michaelides^30^ and Choe et al.^19^ in terms of complexity and system size. We make an attempt to quantify the contribution of the vehicular mechanism and hopping mechanism by comparing the simulation data to the available data on the EOD coefficient and evaluate our model. We expect that our model would perform reasonably well for low water uptakes (λ = 5), since the dominant proton transport mechanism is apparently vehicular transport. At higher water contents, deviations from the computed EOD coefficients and experimental results are expected due to proton hopping.^19,25^

In the second part, we investigate the thermodynamic properties of water inside Nafion 117 as a function of water content. Bai et al.^4^ used calorimetric measurements to obtain physical properties of Nafion at different water uptakes, and analyzed the Gibbs free energy of water uptake in the membrane, leading to the calculation of the partial molar volume of water inside the membrane. These authors also measured densities of different PEM including Nafion membranes and 3M PFSAs. Partial molar volumes of water were obtained from density measurements at different concentrations. Based on the values of the partial molar volumes and enthalpy measurements, the interactions between the polymer and water were investigated. It was observed that strong interactions exist between the water and sulfonate groups at low water contents (λ < 2).^4^ At low water contents, the density of water was found to be higher compared to the density of pure liquid water in the bulk at the same temperature. This is equivalent to a lower partial molar volume of water. For λ > 6, the measured partial molar volume of water shows a plateau around 18 cm^3^ mol^–1^ which is similar to the molar volume of liquid water (18.07 cm^3^ mol^–1^ at *T* = 298.15 K and *P* = 1 atm^31^). It was concluded that, upon an increase in water uptake, the interactions between the side chains of the Nafion membrane are weakened, leading to an increase in volume.^4^ One of the main findings of the study by Bai et al.^4^ is that the partial molar volume analysis is considered complementary when combined with other thermodynamic measurements and studies of the membrane morphology. In this study, we perform a similar analysis from a molecular simulations perspective and check the consistency between the two observations. This also allows us to analyze water–polymer interactions using a physically based model.

Ensemble fluctuations are used to compute the partial molar volume and partial molar excess enthalpy of water in Nafion membranes at different water uptakes.^32,33^ The partial molar enthalpy of water is computed by adding the reference enthalpy, from thermodynamic tables,^34^ to the partial molar excess enthalpy of water computed from simulations. The composition range of water was selected such that the response of the system was in the linear regime. It is important to note that partial molar properties are thermodynamic properties, and the discussion on different transport mechanisms of water do not affect the linearity in this case. A partial molar property *x* of component *i* in a mixture can be defined as ,^34,35^ where *X* is the corresponding extensive property. An alternative way of computing the partial molar properties is using the composition-weighted sum of partial molar properties of the constituent components in the system, namely, .^32,33^ In this paper, we use the second approach to compute the partial molar properties of water. To the best of our knowledge, partial molar properties of water in Nafion membranes were not previously studied using linear regression on raw simulation trajectory data from molecular dynamics.

The rest of this paper is organized as follows: In section 2, simulation details are provided. The structure of Nafion, system sizes, water uptakes, temperatures, and electric fields used in the simulations are specified in this section. The results are presented and discussed in section 3. In this section, the effect of the strength of the electric field and the liquid structure (as characterized by the radial distribution functions, RDFs) is discussed. It is shown that, even for large electric fields, the position of the first and second peaks of the RDFs do not change. The EOD coefficient of water is obtained by computing the average velocities of water and hydronium from the simulations. It is shown that the computed EOD coefficient of water for λ ≤ 5 is within the range of the available experimental data. For λ > 5, the EOD coefficient increases linearly with λ. This may be in part due to the hopping mechanism which is not explicitly included in our classical model. At all values of λ, the partial molar volumes and partial molar excess enthalpies of water were obtained from the molecular simulations. Similar to the experimental data of Bai and Siepmann,^36^ a bulklike behavior of water in Nafion for λ > 5 can be concluded based on the thermodynamic properties of water. Our findings are summarized in Section 4.

## 2 Model and Simulation Details

Classical all-atom force fields are commonly used for Nafion membranes to study the morphology of the membrane, size distribution of water clusters, and transport properties of water molecules and hydronium ions.^1,3,12,37−42^ The model used in this work is a classical all-atom model developed and validated by Lyulin and Sengupta et al.^3,12,37^ Sengupta and Lyulin^37^ used this model to study the structure of Nafion, pairwise interactions between sulfonic sites and water molecules/hydronium ions, transport properties, cluster size distribution of water molecules, diffusion coefficients, and effect of the degree of deprotonation on the structure of hydrated Nafion membranes. This interaction model is a combination of polymer consistent (PCFF)^43^ and COMPASS^44^ force fields.^3^ The PCFF was used for the parameters of the different energy terms in the Class II force field.^45,46^ The COMPASS force field was used to assign the partial charges in the system. The simulation boxes were constructed using the Amorphous Cell module of Material Studio.^47^

The initial configurations were equilibrated by Lyulin et al.^3^ and taken directly from that work. As shown in Figure 1, *m* = 10 represents the degree of polymerization, and the number of repeat units [−CF_2_–CF_2_−]_*n*_ is *n* = 7 corresponding to EW = 1100 g/mol_HSO_3__. In all simulations, 20 Nafion chains are present in the simulation box, and the number of sulfonic acid sites per Nafion chain is *N*_sul_ = 10. The total number of sulfonic acid sites are *N*_SO_3_^–^_ = *N*_H_3_O^+^_ = 200. The number of water molecules not carrying a proton (so excluding hydronium ions) in every system equals *N*_H_2_O_ = 200 × (λ – 1). The total system size ranges from 16 840 atoms (at λ = 5) to 25 840 atoms (at λ = 20). In Table 2, the number of molecules and ions of each species for each λ, and the average volumes at 330, 360, and 420 K are provided. For these typical system sizes, the simulation time may be considerable depending on the computing power.

**Table 2:** Number of Molecules/Ions of Every Species in the Hydrated Nafion System for Every λ^a^

λ	*N*_H_2_O_	*N*_H_3_O^+^_	*N*_Nafion-chains_	*N*_SO_3_–_	⟨*L*⟩_*T*=330K_	⟨*L*⟩_*T*=360K_	⟨*L*⟩_*T*=420K_
5	800	200	20	200	58.14	58.32	58.85
10	1800	200	20	200	60.81	61.10	61.81
15	2800	200	20	200	63.37	63.74	64.59
20	3800	200	20	200	65.78	66.22	67.12

a The average box size ⟨*L*⟩, in [Å], for every temperature is obtained from the average volume.

The molecular dynamics simulations in this study are performed on a periodic system, in all directions, with no interfacial resistance or effects (no boundary edges). This may lead to an overestimation of the EOD coefficient obtained from the simulations. All molecular dynamics simulations are performed using the LAMMPS sorfware package.^48,49^ The velocity-Verlet algorithm^50,51^ is used with an integration step of 1 fs. The Nosé–Hoover thermostat is used both for simulations in the NVT ensemble and NPT ensemble. The Lennard–Jones interactions are computed using the 6/9 functional form with a cutoff of 10 Å. The PPPM method is used to compute the electrostatic interactions beyond the cutoff of 15 Å for the Coulomb potential. In the Supporting Information, a typical input file for LAMMPS is provided for λ = 5 at *T* = 330 K. This system contains 800 water molecules, 200 hydronium ions, and 20 Nafion chains (as indicated in Table 2), and the force field mentioned above is used. The system is equilibrated at *P* = 1 bar.

In all simulations, every single proton is attached to a water molecule, making a hydronium (H_3_O^+^) ion. All systems are charge neutral which means that the number of sulfonic acid groups and hydronium ions are equal (*N*_SO_3_^–^_ = *N*_H_3_O^+^_). The water uptake in the simulation box is computed from
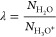
3Since a classical model is used that does not include proton hopping, every proton is fixed to a water molecule. This means that using this model the EOD coefficient (eq 2) is defined as
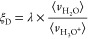
4in which ⟨*v*_H_2_O_⟩ and ⟨*v*_H_3_O^+^_⟩ are the average velocities of the Nafion water and hydronium from the simulation trajectory. Four different hydration levels corresponding to λ = [5, 10, 15, 20] are simulated.

To compute the EOD coefficient using eq 4, the average velocities of water molecules and Nafion ions are sampled from the simulation trajectory under the influence of an external electric field. Ideally, one needs to impose a small electric field corresponding to a typical experimental setup. However, running simulations at weak electric fields results in collecting noise when sampling average velocities in eq 4. To avoid this sampling problem, stronger electric fields are imposed on the system. Four different electric fields were imposed on the system, namely, *e* = [0.02, 0.05, 0.075, 0.100] V/Å. It is important to note that the applied electric fields in the simulation are much stronger than those in a typical experiment. This is typically the case in nonequilibrium molecular dynamics where imposed gradients are much larger than those used in experiments.^52^ As shown in section 3, due to the linear response of the velocities to the electric field, the ratio between the velocities in eq 4 is constant within the statistical uncertainty. This means that for the selected range of the electric fields, the EOD coefficient for every λ is independent of the magnitude of the electric field.

Every electric field is imposed on two independent configurations and along a single axis in the *x*, *y*, or *z* direction in independent simulations. This means that six independent simulations are performed for every value of the electric field. The EOD coefficient (in eq 4) is computed for every electric field. For every λ, the EOD coefficient is averaged over all electric fields at every temperature. Using two independent configurations improves the statistics and imposing the electric field along different axes allows one to observe any directional dependence of EOD coefficient. For every λ, simulations of 175 ns were performed for every electric field at *T* = [330, 360, 420] K. The ratio between the average velocities in eq 4 was extrapolated to the limit of the electric field approaching zero.

To compute partial molar properties (i.e., the change of an extensive quantity with the number of molecules while keeping the temperature and pressure constant) of water in the hydrated Nafion, at every λ, different numbers of water molecules ranging from *N*_water_ = 25 to *N*_water_ = 125 were removed from the initial equilibrated configurations^3^ in steps of 25 molecules. The compositional change is selected such that the energetic and volumetric response of the system remains in the linear regime. This means the change in the volume or total energy of the system has a linear behavior in the compositional range where water molecules are added or removed. To check for possible changes in the structure of the system, the RDFs of the system were computed at different concentrations of water. Simulations were performed in the NPT ensemble. The new configurations, after removing *N*_water_ molecules, were equilibrated for 5 ns, and the production runs were performed for 20 ns. For each water concentration, the fluctuations in volume and total enthalpy of the system were recorded from the simulation trajectories. Linear regression was performed on the total enthalpy and volume of the system as functions of the number of water molecules.^32,33,53,54^ This leads to computation of partial molar excess enthalpy and partial molar volumes of water, respectively. The reference state for the partial molar enthalpy of water can be found in thermodynamic databases or software such as JANAF tables^55,56^ or REFPROP.^57^ Note that it is assumed that partial molar properties for water are composition-independent in the composition range selected for water.^33^ This will be validated in section 3. Pure liquid water was also simulated in the NPT ensemble using the same water model (PCFF parameters) to compute the molar volume of water at atmospheric pressure and at *T* = 330 K and *T* = 360 K. Every simulation box contained 1000 water molecules. The rest of the simulation details are identical to those for the Nafion systems described above. The results are used to compare the partial molar properties of water in Nafion to the molar properties of liquid water. This indicates from which water contents, water in Nafion has a more comparable behavior to the bulk liquid phase.

## 3 Results and Discussion

**Figure 4:**
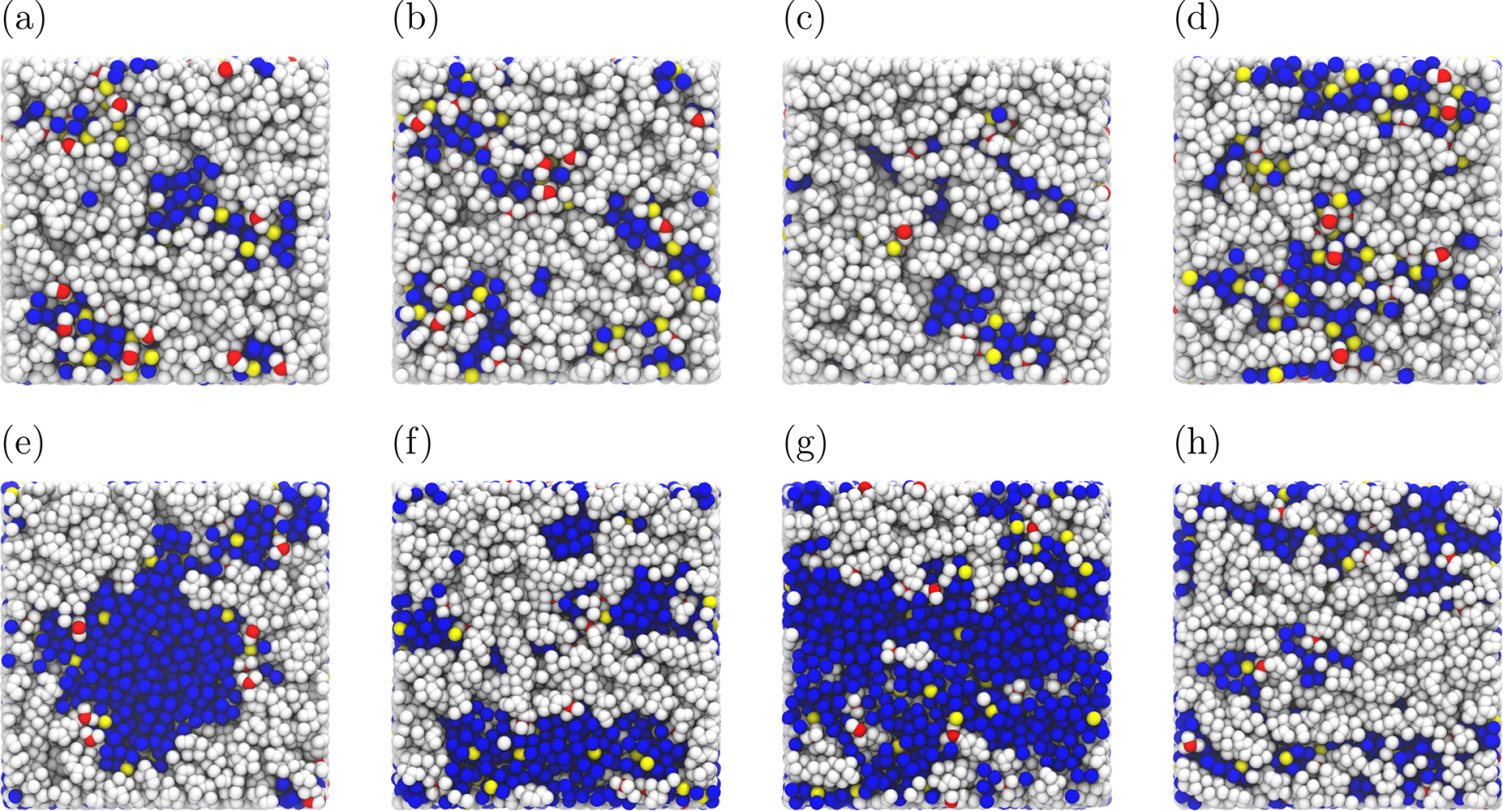
Typical snapshots of hydrated Nafion for two water uptakes (a–d) λ = 5 and (e–h) λ = 20. For snapshots from (a) to (d), and snapshots from (e) to (h), the corresponding strengths of the electric field are *E* = [0, 0.05, 0.075, 0.10] V/Å, respectively. The atoms in the Nafion are shown in white, except for the sulfur atom in the sulfonic acid site (colored red). The water molecules are shown in blue (only oxygen atoms), and the hydronium ions are shown in yellow (only oxygen atoms). For the purpose of this visualization, the hydrogen atoms in water and hydronium are not shown.

**Figure 5:**
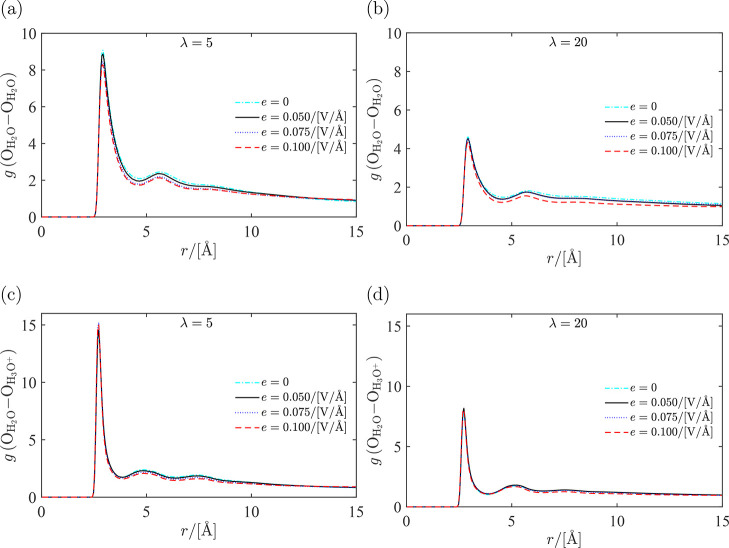
Radial distribution functions for water and hydronium in hydrated Nafion computed for different electric fields imposed on the system at *T* = 330 K and water uptake λ = 5: (a) water–water and (c) water–hydronium; and *T* = 330 K and water uptake λ = 20: (b) water–water and (d) water–hydronium. At every water uptake, electric fields were imposed ranging from 0 to 0.1 V/Å.

**Figure 6:**
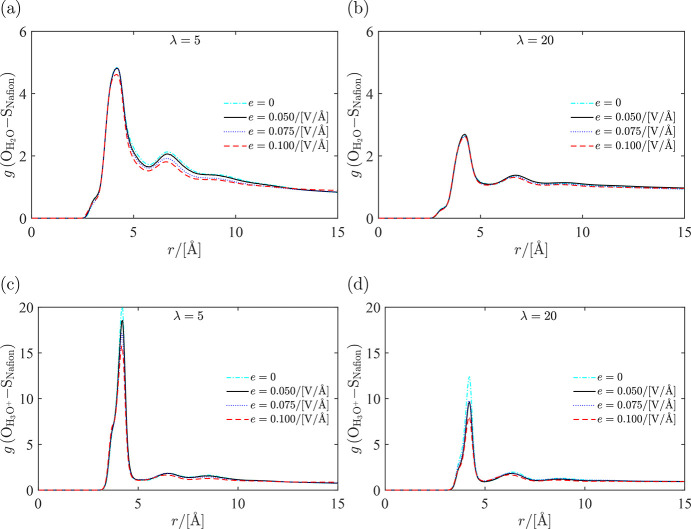
Radial distribution functions for water, hydronium, and the sulfur atom in hydrated Nafion computed for different electric fields imposed on the system at *T* = 330 K and water uptake λ = 5: (a) water–sulfur and (c) hydronium–sulfur; and *T* = 330 K and water uptake λ = 20: (b) water–sulfur and (d) hydronium–sulfur. At every water uptake, electric fields were imposed ranging from 0 to 0.1 V/Å.

Typical snapshots of the hydrated Nafion for λ = 5 and λ = 20 and for electric fields *E* = [0, 0.05, 0.075, 0.10] V/Å are shown in Figure 4. To study the influence of the electric field on the structure of the system, the RDFs for different groups of atoms in the systems are computed and shown in Figures 5 and 6. The resulting coordination numbers are shown in Figures S5 and S6 of the Supporting Information. In Figure 5, the RDFs for water–water and water–hydronium are shown for λ = 5 and λ = 20. The oxygen atoms in water molecules and hydronium ions are used to represent the water molecule and hydronium ion, respectively. It is observed that despite the presence of a strong electric field, the structure of the liquid does not significantly change. The coordinates of the first and second peaks do not change noticeably. For λ = 5, the effect of the electric field on the first peak is more strongly influenced by the imposed electric field compared to λ = 20. At low water uptakes, water is most likely bound to Nafion side chains. Increasing the magnitude of the electric field can influence the interactions between the water molecules and Nafion leading to a more pronounced change in the first peak of the RDF. At high water uptakes, more free volume is occupied by water molecules in which water molecules less bound to the Nafion side chains (bulklike behavior^4^). This is consistent with the observation in Figure 5 that the first peak of the RDF for λ = 20 is less sensitive to changes in the electric field compared to λ = 5. The higher first peak for λ = 5 compared to λ = 20 indicate a strong phase separation between the hydrophobic part of the membrane, water molecules, and hydronium ions. As it will be shown later, the partial molar volume of water is lower for λ = 5 and approaches that of bulk water by increasing the water content. This higher density of the hydrophilic region for low water contents leads to higher peaks in the RDFs. It is observed in Figure 5 that the fist peak of the RDF for hydronium-water is higher at λ = 5 compared to that at λ = 20. At low water uptakes, water and hydronium are most likely closer to the side chains, while at higher water uptakes water molecules and hydronium ions can move more freely and further away from the side chains in the free regions occupied by water. This leads to a difference in the height of the first peak of the RDFs. From Figure S5 in the Supporting Information, it becomes clear that the coordination number of H_2_O and H_3_O^+^ around water depends on λ but not on the magnitude of the electric field. In Figure 6, the RDFs for water–sulfur and hydronium–sulfur are shown for λ = 5, and λ = 20. For hydronium-sulfur, it is observed the first peak decreases significantly with the increasing electric field. This indicates that with increasing electric field, hydronium ions have weaker interactions with the sulfonic sites, and can move more freely in the system. This observation can also be seen in the coordination numbers as shown in Figure S6 in the Supporting Information. The interactions between water and sulfonic sites are less influenced by the electric field especially for λ = 20. This indicates bulklike behavior of water at higher concentrations as concluded by Bai et al.^4^

**Figure 7:**
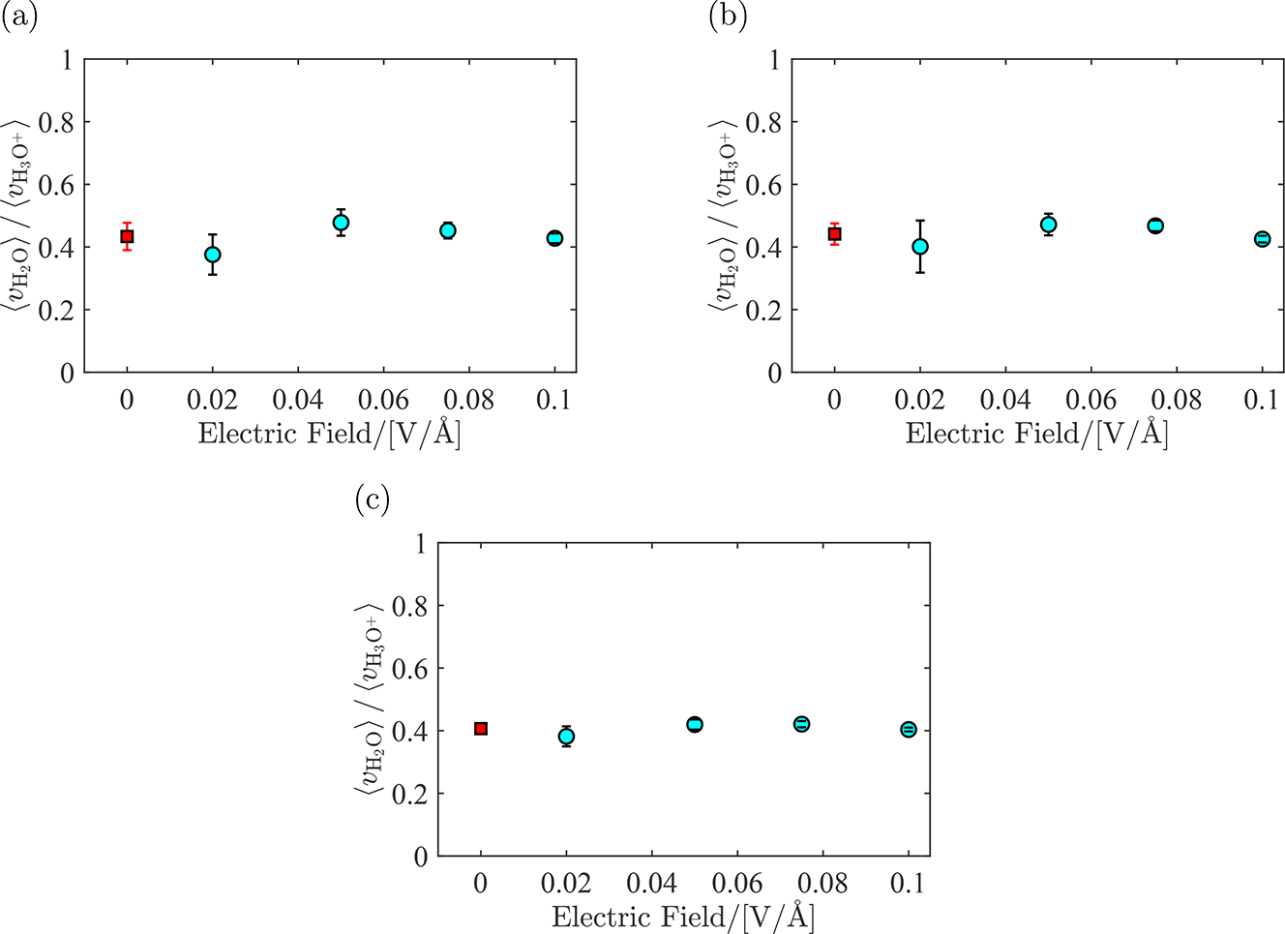
Ratio between the velocities of water and hydronium (circles) for λ = 5 at different magnitudes of the electric field imposed on the simulation box at (a) *T* = 330 K, (b) *T* = 360 K, and (c) *T* = 420 K. Squares are the average over the ratio between the velocities. Raw data are provided in Table S1 in the Supporting Information. For λ = 10 and λ = 15, raw data are provided in Tables S2 and S3 in the Supporting Information.

**Figure 8:**
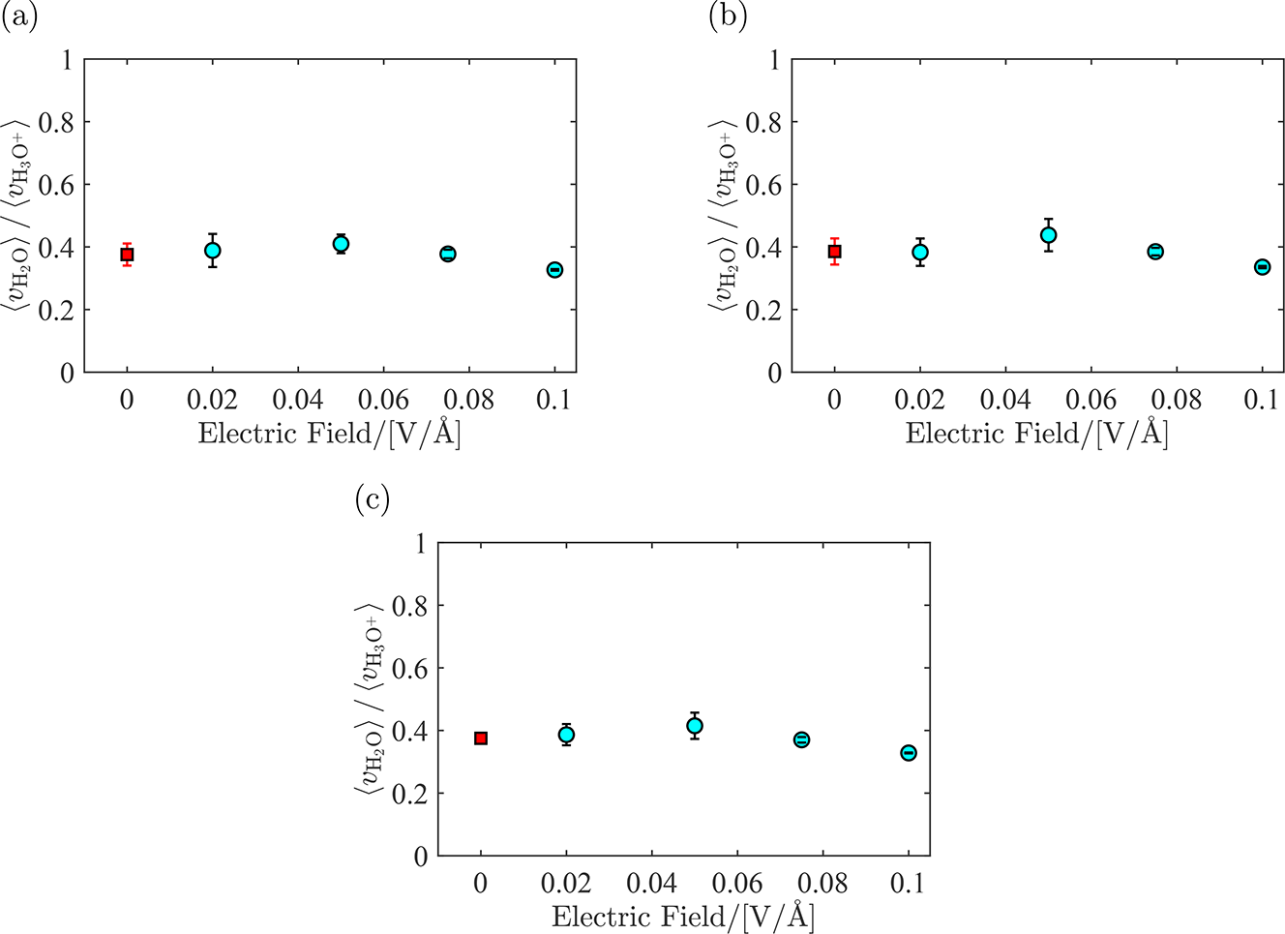
Ratio between the velocities of water and hydronium for λ = 20 at different magnitudes of the electric field imposed on the simulation box at (a) *T* = 330 K, (b) *T* = 360 K, and (c) *T* = 420 K. Squares are the average over the ratio between the velocities. Raw data are provided in Table S4 in the Supporting Information.

The ratio between average velocities of water and hydronium, ⟨*v*_H_2_O_⟩/⟨*v*_H_3_O^+^_⟩, is computed from molecular dynamics simulations in LAMMPS,^48,49^ in the presence of an electric field, for every λ. One would expect larger uncertainties in this ratio at low electric fields due to lower signal-to-noise ratios. In Figure 7, this ratio is shown for λ = 5 at *T* = [330, 360, 420] K for *e* = [0.02, 0.05, 0.075, 0.100] V/Å. Each point in this figure is the average of six independent simulations, as explained in Section 2, and the uncertainties are the standard deviations from block averaging. Simulation results show that within the error bars, the average velocities of H_2_O and H_3_O^+^ scale linearly with the electric field. This means that the ratio between these average velocities is constant. This can be verified both from Figures 6 and 8 where the velocity ratio between water and hydronium is constant and independent of the electric field (within the statistical uncertainties). Therefore, for computing the EOD coefficient, the ratio ⟨*v*_H_2_O_⟩/⟨*v*_H_3_O^+^_⟩ was averaged over all electric fields. The corresponding figures for λ = 10 and λ = 15 are provided in Figures S1 and S2 in the Supporting Information. It is observed that the ratio between the velocities of water and hydronium for all λ is approximately 0.4. The raw velocity data for all temperatures and water uptakes are provided in Tables S1–S4 in the Supporting Information.

**Figure 9:**
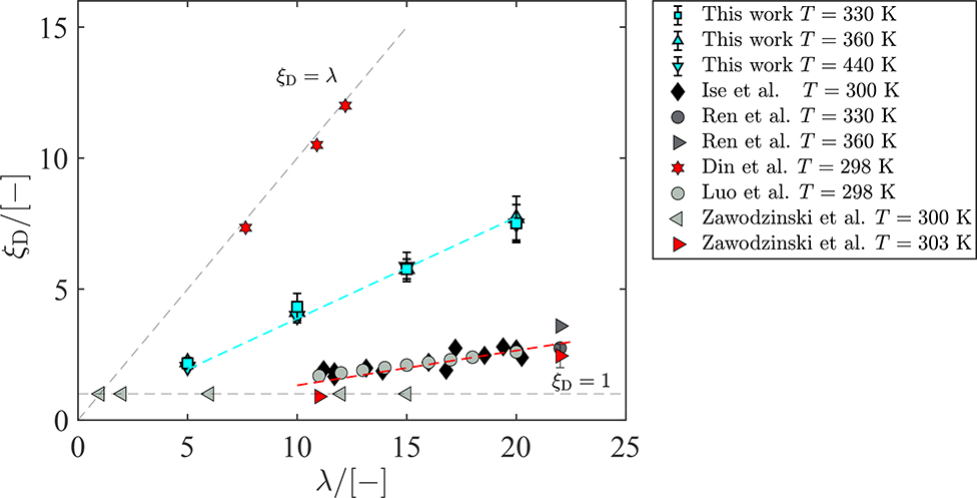
Electro-osmotic drag coefficient obtained from molecular dynamics simulations in LAMMPS^48,49^ as a function of the water uptake for *T* = 330 K, *T* = 360 K, and *T* = 420 K. Error bars are uncertainties obtained from block averaging of 6 independent simulations. The electro-osmotic drag coefficients from experiments are obtained from refs..^2,8,58−60^ The electro-osmotic drag by Din and Michaelides^30^ is computed from molecular dynamics. The slopes of the fitted lines for the simulation data and experimental data are 0.40 and 0.13, respectively. Raw data obtained from molecular dynamics simulations in this work are provided in Table S5 in the Supporting Information.

The EOD coefficient of water in Nafion for every water uptake is obtained using eq 4 and the results are shown in Figure 9. Although there is a scattering in experimental data, it can be concluded from Figure 9 that the actual EOD coefficient is most likely closer to the line ξ_D_ = 1 compared to ξ_D_ = λ. Comparing the EOD coefficients obtained in this work to the results obtained by Din and Michaelides^30^ shows that using a more elaborate classical model significantly improves the computed EOD coefficient. It is observed that the EOD coefficient of water obtained from our molecular dynamics simulations changes linearly with λ, in sharp contrast to experimental results of Zawodzinski et al.^58^ Zawodzinski et al. found that the EOD coefficient in vapor equilibrated Nafion is constant (ca. 1.0) and for liquid equilibrated Nafion (high water uptakes) it is ca. 2.5. The results from ab initio molecular dynamics by Choe et al.^19^ are in good agreement with the reported EOD coefficients by Zawodzinski et al.^58^ for vapor equilibrated Nafion. However, at higher water contents, other experimental data^2,8,59,60^ predict higher values for the EOD coefficient as shown in Figure 9. It is argued in refs (15) and (19) that not considering proton hopping in the model leads to an overestimation of the drag coefficient at high water uptakes. This agrees well with the fact that our model overpredicts the EOD coefficient for λ between 10 and 20. However, at λ = 5, it can be argued that the deviation is significantly smaller and the EOD coefficient is within the range of experimental data. This may lead to the conclusion that our model can predict the vehicular transport of proton at low water uptakes where proton hopping does not dominate (λ < 5).

The linear behavior of the EOD coefficient of water from our molecular dynamics simulations agrees with the data by Ise et al.^2^ Both results show a monotonic increase in the EOD coefficient with the increasing λ. The monotonic increase of the EOD coefficient agrees in general well with the general observation by Kusoglu and Weber^1^ when considering all collected experimental data. The linear fit to the experimental data in Figure 9 leads to a slope of ca. 0.13 (ξ_D_ ≈ 0.13λ), while a linear fit to simulation data leads to a slope of ca. 0.40 (ξ_D_ ≈ 0.40λ). The difference in EOD coefficient is larger for higher water content and can be approximately quantified using the slope of the fitted lines. This difference is partly due to the absence of the Grotthuss mechanism in the simulations. We also observe that, within the error bars, the EOD coefficient obtained from the molecular dynamics simulations does not considerably change with temperature for different values of λ. LaConti et al.^23,61^ also reported a linear increase of the EOD coefficient between 0 and 5, corresponding to dry and fully hydrated membrane, with no temperature dependence.^2^ Another factor which may have contributed to larger values of the EOD coefficient in this work may be due to the absence of interfacial resistance (entrance or exit effects), as present in experiments..^62,63^ Cheah et al. show that the water flux into and out of the membrane is affected by the interfacial transport between the membrane and the vapor phase.^62^ Due to the periodic boundary conditions in our simulations, these interfacial effects are not captured. The interfacial resistance is reduced with liquid water presence at the interface of the membrane instead of vapor.^58,63,64^

**Figure 10:**
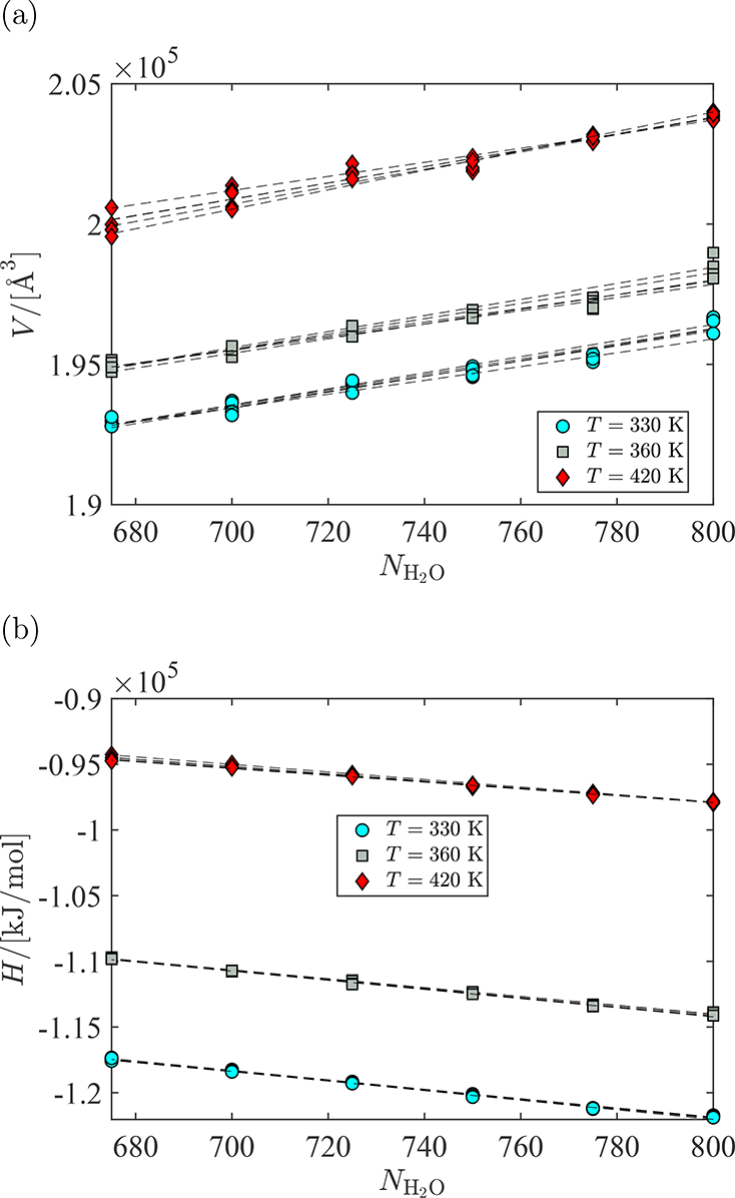
(a) Volume and (b) specific enthalpy of the Nafion system at a water uptake λ = 5 (*N*_water_ = 800) and corresponding systems, where between 25 and 125 water molecules are removed while keeping the pressure and temperature constant. This range is selected such that compositional changes throughout the simulation trajectory remains linear. The trajectories are divided into five blocks to calculate the uncertainties. The partial molar volumes and partial molar enthalpies of water at every temperature are obtained by calculating the slope for every block and averaging over the slopes. Dashed lines are the regression lines shown as a guide for the reader.

The fluctuations in volume and total enthalpy of the system as a function of the number of water molecules are used to compute the partial molar volume and partial molar enthalpy of water in Nafion. In Figure 10, this is shown for the hydrated Nafion at λ = 5. The changes in enthalpy and volume of the system are linear with respect to the changes in the number of water molecules. For every composition, the simulation trajectories were divided into five blocks after equilibration. For every block, linear regression is used to compute the slope at every temperature. The partial molar volume and partial molar enthalpy of water are computed from block averaging of the slopes.

**Figure 11:**
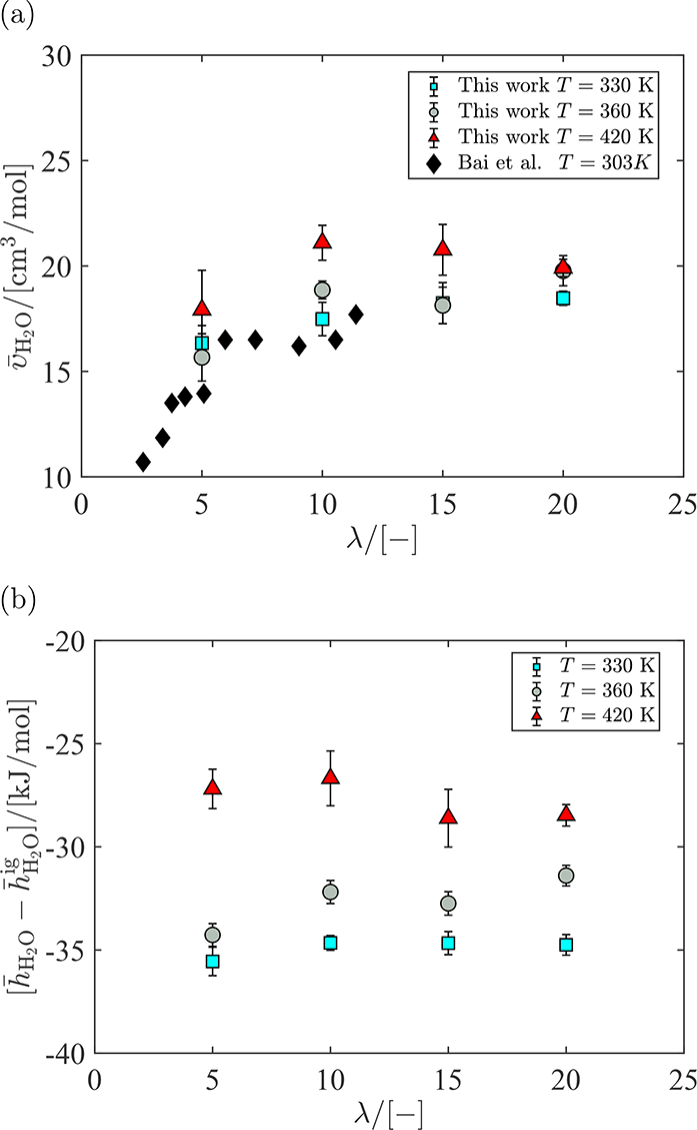
(a) Partial molar volumes of water and (b) partial molar enthalpies of water as a function of water uptake in the Nafion. Uncertainties are calculated using block averaging (5 blocks). The molar properties of pure water computed using the CFF force field are (in units of cm^3^/mol and kJ/mol, respectively) *v̅*_H_2_O_ = 19.76(2), *h̅*_H_2_O_ – *h̅*_H_2_O_^ig^ = −28.29(1) at *T* = 330 K, *v̅*_H_2_O_ = 20.45(1), *h̅*_H_2_O_ – *h̅*_H_2_O_^ig^ = −26.57(1) at *T* = 360 K. At *T* = 420 K, the saturation pressure for the liquid phase is higher than 1 atm, and therefore, the molar properties of pure liquid water at *T* = 420 K are not considered here. Raw data are provided in Table S6 in the Supporting Information. The term *h̅*_H_2_O_^ig^ denotes the ideal gas part of the enthalpy of water.

The partial molar volume of water computed at *T* = [330, 360, 420] K at water uptake 5 ≤ λ ≤ 20 is shown in Figure 11a. To compare the performance of the water model in pure water with that in the hydrated Nafion, the molar volume and molar excess enthalpy of pure water was also computed using the same water model, and the results are provided in the caption of Figure 11. The partial molar volume of water is lower at λ = 5 compared to higher water uptakes. This indicates a higher density at λ = 5 and a strong and favorable interaction between the water molecules and the sulfonic sites in the side chain of the membrane. For λ = 10 to λ = 20, the partial molar volume of water reaches a plateau. This is in excellent agreement with the observation of Bai et al.,^4^ reporting the plateau for λ > 6. The experimental data from ref (4) are also provided in Figure 11. At higher water uptakes, water is less confined by sulfonate groups, leading to an increased partial molar volume of water.^1,4^ This means that, for large water uptakes, the morphology of the membrane allows channels in which water has a similar behavior to the bulk liquid phase.^4^ At all temperatures, it is observed that the value of the partial molar volume of water approaches to the molar volume of bulk water with increasing λ. The RDFs of the system for compositions close to λ = 5 and λ = 20 are shown in Figures S3 and S4 in the Supporting Information.

In Figure 11b, the partial molar enthalpy of water with respect to the ideal gas reference state (*h̅*_H_2_O_^ig^) is shown as a function of λ. From Figure 11b, it is observed that the enthalpy change of water uptake is negative, indicating an exothermic process. This is in agreement with the experimental work by Bai et al.^4^ Further consideration of the results in Figure 11b shows that the partial molar enthalpy of water is smallest at λ = 5 and reaches a plateau for λ ≥ 10. Compared to molar enthalpy of pure water, the partial molar enthalpy of water is lower. Lower partial molar volumes and enthalpies of water compared to the molar properties of pure water show that the model can capture the favorable interactions between the water and the hydrophilic side chain of Nafion.

## 4 Conclusions

The electro-osmotic drag (EOD) coefficient of water in Nafion 117 (EW = 1100 g/mol_HSO_3__) is computed using molecular dynamics. The physical model used in this work^3^ is an all-atom force field which is a combination of polymer consistent and COMPASS force fields. Since this is a classical model, proton hopping (Grotthuss mechanism)^27^ is inherently absent in the simulations. Only the vehicular mechanism is captured in the model which is the dominant mechanism at low water contents.^15,19^ Consequently, the model overestimates the EOD coefficient at high water uptakes due to the lack of proton hopping while the performance of the model is much better at lower water uptakes. We computed the EOD coefficient of water at different water contents ranging from 5 to 20. To compute the ratio between the velocities of water and hydronium ions in the system, electric fields of varying strength were applied to the system. It was found that the velocities of water and hydronium scaled linearly with the electric field which means that the ratio is constant. The ratio between the velocities of water and hydronium is therefore obtained by averaging over all electric fields. As shown in the review paper of Kusoglu and Weber,^1^ the reported values of the EOD coefficient of water from experiments shows scattering. Similar to the results obtained by Ise et al.,^2^ the EOD coefficient obtained from molecular dynamics simulations shows a monotonic increase with increasing water uptake in the membrane. At λ = 20, the EOD coefficient is ca. 3 times higher compared to the experimental data. At λ = 5, the value of the EOD coefficient is ca. 2 which is within the same range as the experimental data. This indicates that the model captures the vehicular mechanism well, and can predict the EOD coefficient reasonably well where the proton hopping is not dominant. In future work, it would be interesting to see the effect of the residence times between water and hydronium and investigate how this influences the EOD. Since the interfacial resistance between the membrane and the liquid/vapor is not considered in the simulations, this may also lead to a deviation of the computed EOD coefficient compared to experimental data. The thermodynamic properties of the hydrated Nafion system are captured well using this model. The same trend with the change in computed partial molar volumes of water from molecular simulations is observed as in the experimental results by Bai et al.^4^ The results show that the partial molar volume of water is lowest at λ = 5 and shows a plateau when increasing λ. It can be concluded that, at lower water uptakes, the favorable interactions of water and sulfonic sites result in a higher density for water (compared to pure liquid water). This observation provides an important correlation between thermodynamic properties of water and the morphology of the hydrated Nafion. Computed partial molar enthalpies of water in Nafion show that water absorption, at the simulation conditions, is an exothermic process. This is in agreement with the experimental observation of Bai et al.^4^ The enthalpy of water in hydrated Nafion also increases with the increase of water uptake. This observation based on the partial molar enthalpy of water also confirms that the model can correctly capture the bulklike behavior of the model and the presence of free water in the membrane. The results show that the models used here capture the thermodynamics quite well while a discrepancy arises in the dynamic properties of water transport when the proton hopping mechanism is not considered. This discrepancy becomes significant at higher water uptakes. It is so far not clear how the proton hopping in a force field-based classical model can be captured. This can be the topic of future research.

## Supporting Information Available

Raw simulation data including the ratio between average velocities of water hydronium at different temperatures, water contents, and electric fields (corresponding to Figures 7 and 8), electro-osmotic drag coefficient obtained from molecular dynamics simulations in this work (corresponding to Figure 9), partial molar volumes and partial molar excess enthalpies (corresponding to Figure 11), radial distribution functions for water and hydronium at different water uptakes around λ = 5 and λ = 20 (no imposed electric field), coordination numbers correcponding to Figures 5 and 6 (PDF)
Typical input file for LAMMPS is provided for λ = 5 at *T* = 330 K (ZIP)

## References

[ref1] Kusoglu A.; Weber A. Z. New Insights into Perfluorinated Sulfonic-Acid Ionomers. Chem. Rev. 2017, 117, 987–1104. 10.1021/acs.chemrev.6b00159. 28112903

[ref2] Ise M.; Kreuer K.; Maier J. Electroosmotic drag in polymer electrolyte membranes: an electrophoretic NMR study. Solid State Ion 1999, 125, 213–223. 10.1016/S0167-2738(99)00178-2.

[ref3] Lyulin A. V.; Sengupta S.; Varughese A.; Komarov P.; Venkatnathan A. Effect of Annealing on Structure and Diffusion in Hydrated Nafion Membranes. ACS Appl. Polym. Mater. 2020, 2, 5058–5066. 10.1021/acsapm.0c00875.

[ref4] Bai Y.; Schaberg M. S.; Hamrock S. J.; Tang Z.; Goenaga G.; Papandrew A. B.; Zawodzinski T. A. Jr Density measurements and partial molar volume analysis of different membranes for polymer electrolyte membrane fuel cells. Electrochim. Acta 2017, 242, 307–314. 10.1016/j.electacta.2017.04.048.

[ref5] Okada T.; Xie G.; Gorseth O.; Kjelstrup S.; Nakamura N.; Arimura T. Ion and water transport characteristics of Nafion membranes as electrolytes. Electrochim. Acta 1998, 43, 3741–3747. 10.1016/S0013-4686(98)00132-7.

[ref6] Rohland B.; Eberle K.; Ströbel R.; Scholta J.; Garche J. Electrochemical hydrogen compressor. Electrochim. Acta 1998, 43, 3841–3846. 10.1016/S0013-4686(98)00144-3.

[ref7] Ströbel R.; Oszcipok M.; Fasil M.; Rohland B.; Jörissen L.; Garche J. The compression of hydrogen in an electrochemical cell based on a PEM fuel cell design. J. Power Sources 2002, 105, 208–215. 10.1016/S0378-7753(01)00941-7.

[ref8] Zawodzinski T. A.; Springer T. E.; Davey J.; Jestel R.; Lopez C.; Valerio J.; Gottesfeld S. A Comparative Study of Water Uptake By and Transport Through Ionomeric Fuel Cell Membranes. J. Electrochem. Soc. 1993, 140, 1981–1985. 10.1149/1.2220749.

[ref9] Mauritz K. A.; Moore R. B. State of Understanding of Nafion. Chem. Rev. 2004, 104, 4535–4586. 10.1021/cr0207123. 15669162

[ref10] Kee B. L.; Curran D.; Zhu H.; Braun R. J.; DeCaluwe S. C.; Kee R. J.; Ricote S. Thermodynamic Insights for Electrochemical Hydrogen Compression with Proton-Conducting Membranes. Membranes 2019, 9, 77 10.3390/membranes9070077. PMC6680696 31266218

[ref11] Sdanghi G.; Maranzana G.; Celzard A.; Fierro V. Review of the current technologies and performances of hydrogen compression for stationary and automotive applications. Renew. Sust. Energy Rev. 2019, 102, 150–170. 10.1016/j.rser.2018.11.028.

[ref12] Sengupta S.; Lyulin A. V. Molecular Modeling of Structure and Dynamics of Nafion Protonation States. J. Phys. Chem. B 2019, 123, 6882–6891. 10.1021/acs.jpcb.9b04534. 31306017 PMC6691399

[ref13] Bouwman P. J.; Konink J.; Semerel D.; Raymakers L.; Koeman M.; Kout W.; Dalhuijsen W.; Milacic E.; Mulder M. J. J. Electrochemical Hydrogen Compression. ECS Trans. 2014, 64, 1009–1018. 10.1149/06403.1009ecst.

[ref14] Rahbari A.; Brenkman J.; Hens R.; Ramdin M.; van den Broeke L. J. P.; Schoon R.; Henkes R.; Moultos O. A.; Vlugt T. J. H. Solubility of water in hydrogen at high Pressures: a molecular simulation study. J. Chem. Eng. Data 2019, 64, 4103–4115. 10.1021/acs.jced.9b00513.

[ref15] Jiao K.; Li X. Water transport in polymer electrolyte membrane fuel cells. Prog. Energy Combust. Sci. 2011, 37, 221–291. 10.1016/j.pecs.2010.06.002.

[ref16] Springer T. E.; Zawodzinski T. A.; Gottesfeld S. Polymer Electrolyte Fuel Cell Model. J. Electrochem. Soc. 1991, 138, 2334–2342. 10.1149/1.2085971.

[ref17] Ionescu V. Water and hydrogen transport modelling through the membrane-electrode assembly of a PEM fuel cell. Phys. Scr. 2020, 95, 034006 10.1088/1402-4896/ab51ee.

[ref18] Dickinson E. J. F.; Smith G. Modelling the Proton-Conductive Membrane in Practical Polymer Electrolyte Membrane Fuel Cell (PEMFC) Simulation: A Review. Membranes 2020, 10 10.3390/membranes10110310. 33126688 PMC7692588

[ref19] Choe Y.-K.; Tsuchida E.; Ikeshoji T.; Yamakawa S.; Hyodo S.-a. Nature of Water Transport and Electro-Osmosis in Nafion: Insights from First-Principles Molecular Dynamics Simulations under an Electric Field. J. Phys. Chem. B 2008, 112, 11586–11594. 10.1021/jp8041878. 18717541

[ref20] Gogoi A.; Reddy K. A.; Mondal P. K. Electro-osmotic flow through nanochannel with different surface charge configurations: A molecular dynamics simulation study. Phys. Fluids 2021, 33, 092115 10.1063/5.0062031.

[ref21] Wang Y.; Liu R.; Liu Z. Nonlinearly induced electro-osmotic flow reversal in charged nanotube: Counter-ions mobility in Stern layer. Int. J. Heat Mass Transfer 2022, 188, 122587 10.1016/j.ijheatmasstransfer.2022.122587.

[ref22] Abdulagatov I. M.; Bazaev A. R.; Bazaev E. A.; Khokhlachev S. P.; Saidakhmedova M. B.; Ramazanova A. E. Excess, partial, and molar volumes of n-alkanes in near-critical and supercritical water. J. Solution Chem. 1998, 27, 731–753. 10.1023/A:1022657607502.

[ref23] Karimi G.; Li X. Electroosmotic flow through polymer electrolyte membranes in PEM fuel cells. J. Power Sources 2005, 140, 1–11. 10.1016/j.jpowsour.2004.08.018.

[ref24] Choi P.; Jalani N. H.; Datta R. Thermodynamics and proton transport in nafion: II. Proton diffusion mechanisms and conductivity. J. Electrochem. Soc. 2005, 152, E123 10.1149/1.1859814.

[ref25] Sellin R.; Mozet K.; Ménage A.; Dillet J.; Didierjean S.; Maranzana G. Measuring electro-osmotic drag coefficients in PFSA membranes without any diffusion assumption. Int. J. Hydrogen Energy 2019, 44, 24905–24912. 10.1016/j.ijhydene.2019.07.076.

[ref26] Pivovar B. S. An overview of electro-osmosis in fuel cell polymer electrolytes. Polymer 2006, 47, 4194–4202. 10.1016/j.polymer.2006.02.071.

[ref27] Agmon N. The Grotthuss mechanism. Chem. Phys. Lett. 1995, 244, 456–462. 10.1016/0009-2614(95)00905-J.

[ref28] Markovitch O.; Agmon N. Structure and Energetics of the Hydronium Hydration Shells. J. Phys. Chem. A 2007, 111, 2253–2256. 10.1021/jp068960g. 17388314

[ref29] Markovitch O.; Chen H.; Izvekov S.; Paesani F.; Voth G. A.; Agmon N. Special Pair Dance and Partner Selection: Elementary Steps in Proton Transport in Liquid Water. J. Phys. Chem. B 2008, 112, 9456–9466. 10.1021/jp804018y. 18630857

[ref30] Din X.-D.; Michaelides E. E. Transport processes of water and protons through micropores. AIChE J. 1998, 44, 35–47. 10.1002/aic.690440106.

[ref31] Wagner W.; Pruß A. The IAPWS formulation 1995 for the thermodynamic properties of ordinary water substance for general and scientific use. J. Phys. Chem. Ref. Data 2002, 31, 387–535. 10.1063/1.1461829.

[ref32] Josephson T. R.; Singh R.; Minkara M. S.; Fetisov E. O.; Siepmann J. I. Partial molar properties from molecular simulation using multiple linear regression. Mol. Phys. 2019, 117, 3589–3602. 10.1080/00268976.2019.1648898.

[ref33] Rahbari A.; Josephson T. R.; Sun Y.; Moultos O. A.; Dubbeldam D.; Siepmann J. I.; Vlugt T. J. H. Multiple Linear Regression and Thermodynamic Fluctuations are Equivalent for Computing Thermodynamic Derivatives from Molecular Simulation. Fluid Phase Equilib. 2020, 523, 112785 10.1016/j.fluid.2020.112785.

[ref34] Sandler S. I. Chemical, Biochemical, and Engineering Thermodynamics, 4th ed.; John Wiley & Sons: Hoboken, NJ, 2006.

[ref35] Sindzingre P.; Ciccotti G.; Massobrio C.; Frenkel D. Partial enthalpies and related quantities in mixtures from computer simulation. Chem. Phys. Lett. 1987, 136, 35–41. 10.1016/0009-2614(87)87294-9.

[ref36] Bai P.; Siepmann J. I. Selective adsorption from dilute solutions: Gibbs ensemble Monte Carlo simulations. Fluid Phase Equilib. 2013, 351, 1–6. 10.1016/j.fluid.2012.08.014.

[ref37] Sengupta S.; Lyulin A. V. Molecular Dynamics Simulations of Substrate Hydrophilicity and Confinement Effects in Capped Nafion Films. J. Phys. Chem. B 2018, 122, 6107–6119. 10.1021/acs.jpcb.8b03257. 29757641 PMC5994720

[ref38] Li Z.-Z.; Chen L.; Tao W.-Q. Molecular dynamics simulation of water permeation through the Nafion membrane. Numer Heat Tr. A-Appl. 2016, 70, 1232–1241. 10.1080/10407782.2016.1230424.

[ref39] Urata S.; Irisawa J.; Takada A.; Shinoda W.; Tsuzuki S.; Mikami M. Molecular Dynamics Study of the Methanol Effect on the Membrane Morphology of Perfluorosulfonic Ionomers. J. Phys. Chem. B 2005, 109, 17274–17280. 10.1021/jp052647h. 16853205

[ref40] Karo J.; Aabloo A.; Thomas J. O.; Brandell D. Molecular Dynamics Modeling of Proton Transport in Nafion and Hyflon Nanostructures. J. Phys. Chem. B 2010, 114, 6056–6064. 10.1021/jp903288y. 20402464

[ref41] Venkatnathan A.; Devanathan R.; Dupuis M. Atomistic Simulations of Hydrated Nafion and Temperature Effects on Hydronium Ion Mobility. J. Phys. Chem. B 2007, 111, 7234–7244. 10.1021/jp0700276. 17518488

[ref42] Jacobson L. C.; Ren X.; Molinero V. Assessing the Effects of Crowding, Pore Size, and Interactions on Electro-Osmotic Drag Coefficients. J. Phys. Chem. C 2014, 118, 2093–2103. 10.1021/jp410910r.

[ref43] Sun H.; Mumby S. J.; Maple J. R.; Hagler A. T. An ab initio CFF93 all-atom force field for polycarbonates. Journal of the American Chemical society 1994, 116, 2978–2987. 10.1021/ja00086a030.

[ref44] Sun H. COMPASS: an ab initio force-field optimized for condensed-phase applications overview with details on alkane and benzene compounds. J. Phys. Chem. B 1998, 102, 7338–7364. 10.1021/jp980939v.

[ref45] Sun H.; Jin Z.; Yang C.; Akkermans R. L.; Robertson S. H.; Spenley N. A.; Miller S.; Todd S. M. COMPASS II: extended coverage for polymer and drug-like molecule databases. J. Mol. Model. 2016, 22, 47 10.1007/s00894-016-2909-0. 26815034

[ref46] Hwang M. J.; Stockfisch T.; Hagler A. Derivation of class II force fields. 2. Derivation and characterization of a class II force field, CFF93, for the alkyl functional group and alkane molecules. J. Am. Chem. Soc. 1994, 116, 2515–2525. 10.1021/ja00085a036.

[ref47] Module C. Material Studio, ver. 7.0; Accelrys Inc., San Diego, CA, 2013.

[ref48] Plimpton S. Fast parallel algorithms for short-range molecular dynamics. J. Comput. Phys. 1995, 117, 1–19. 10.1006/jcph.1995.1039.

[ref49] Thompson A. P.; Aktulga H. M.; Berger R.; Bolintineanu D. S.; Brown W. M.; Crozier P. S.; in ’t Veld P. J.; Kohlmeyer A.; Moore S. G.; Nguyen T. D.; et al. LAMMPS - a flexible simulation tool for particle-based materials modeling at the atomic, meso, and continuum scales. Comput. Phys. Commun. 2022, 271, 108171 10.1016/j.cpc.2021.108171.

[ref50] Frenkel D.; Smit B. Understanding Molecular Simulation: From Algorithms to Applications, 2nd ed.; Academic Press: San Diego, CA, 2002.

[ref51] Allen M. P.; Tildesley D. J. Computer Simulation of Liquids, 2nd ed.; Oxford University Press: Oxford, United Kingdom, 2017.

[ref52] J Evans D.; P Morriss G. Statistical mechanics of nonequilbrium liquids, 2nd ed.; ANU E Press: Canberra, Australia, 2007.

[ref53] Karavias F.; Myers A. L. Isosteric heats of multicomponent adsorption: thermodynamics and computer simulations. Langmuir 1991, 7, 3118–3126. 10.1021/la00060a035.

[ref54] Walpole R. E.; Myers R. H.; Myers S. L.; Ye K. Probability & Statistics for Engineers & Scientists, 9th ed.; Prentice Hall: Boston, 2012.

[ref55] Chase M. W. NIST-JANAF Themochemical Tables, Fourth Edition. J. Phys. Chem. Ref. Data 1998, 4, 1–1951.

[ref56] Chase M. W.; Curnutt J.; Prophet H.; McDonald R.; Syverud A. JANAF thermochemical tables, 1975 supplement. J. Phys. Chem. Ref. Data. 1975, 4, 1–176. 10.1063/1.555517.

[ref57] Lemmon E. W.; Huber M. L.; McLinden M. O. NIST reference fluid thermodynamic and transport properties–REFPROP. NIST standard reference database 2002, 23, ver. 7. https://www.nist.gov/srd/refprop (accessed 2021-07-01).

[ref58] Zawodzinski T. A.; Davey J.; Valerio J.; Gottesfeld S. The water content dependence of electro-osmotic drag in proton-conducting polymer electrolytes. Electrochim. Acta 1995, 40, 297–302. 10.1016/0013-4686(94)00277-8.

[ref59] Ren X.; Gottesfeld S. Electro-osmotic drag of water in poly (perfluorosulfonic acid) membranes. J. Electrochem. Soc. 2001, 148, A87 10.1149/1.1344521.

[ref60] Luo Z.; Chang Z.; Zhang Y.; Liu Z.; Li J. Electro-osmotic drag coefficient and proton conductivity in Nafion^®^ membrane for PEMFC. Int. J. Hydrogen Energy 2010, 35, 3120–3124. 10.1016/j.ijhydene.2009.09.013.

[ref61] LaConti A. B.; Fragala A. R.; Boyack J. R. Proceedings of the Symposium on Electrode Materials and Processes for Energy Conversion and Storage, Vol 77; McIntyre D. E., Srinivasan S., Will E. G., Eds.; 1977, p 354.

[ref62] Cheah M. J.; Kevrekidis I. G.; Benziger J. Effect of Interfacial Water Transport Resistance on Coupled Proton and Water Transport Across Nafion. J. Phys. Chem. B 2011, 115, 10239–10250. 10.1021/jp204785t. 21780814

[ref63] Majsztrik P.; Bocarsly A.; Benziger J. Water Permeation through Nafion Membranes: The Role of Water Activity. J. Phys. Chem. B 2008, 112, 16280–16289. 10.1021/jp804197x. 19053672

[ref64] Majsztrik P. W.; Satterfield M. B.; Bocarsly A. B.; Benziger J. B. Water sorption, desorption and transport in Nafion membranes. J. Membr. Sci. 2007, 301, 93–106. 10.1016/j.memsci.2007.06.022.

[ref65] Breslau B. R.; Miller I. F. A hydrodynamic model for electroosmosis. Ind. Eng. Chem. 1971, 10, 554–565. 10.1021/i160040a003.

[ref66] Zawodzinski T. A. Jr; Derouin C.; Radzinski S.; Sherman R. J.; Smith V. T.; Springer T. E.; Gottesfeld S. Water uptake by and transport through Nafion® 117 membranes. J. Electrochem. Soc. 1993, 140, 1041 10.1149/1.2056194.

[ref67] Mayer K.; Woermann D. Diffusion- and convection-induced transport of nonelectrolytes in aqueous solution across a cation-exchange membrane. J. Membr. Sci. 1997, 127, 35–45. 10.1016/S0376-7388(96)00293-1.

[ref68] Pivovar B. S.; Smyrl W. H.; Cussler E. L. Electro-osmosis in Nafion 117, Polystyrene Sulfonic Acid, and Polybenzimidazole. J. Electrochem. Soc. 2005, 152, A53 10.1149/1.1827599.

[ref69] Verbrugge M. W.; Hill R. F. Transport Phenomena in Perfluorosulfonic Acid Membranes during the Passage of Current. J. Electrochem. Soc. 1990, 137, 1131–1138. 10.1149/1.2086615.

[ref70] Ren X.; Henderson W.; Gottesfeld S. Electro-osmotic Drag of Water in Ionomeric Membranes: New Measurements Employing a Direct Methanol Fuel Cell. J. Electrochem. Soc. 1997, 144, L267–L270. 10.1149/1.1837940.

[ref71] Ren X.; Gottesfeld S. Electro-osmotic Drag of Water in Poly(perfluorosulfonic acid) Membranes. J. Electrochem. Soc. 2001, 148, A87 10.1149/1.1344521.

[ref72] Pivovar B. S.; Hickner M.; Zawodzinski T. A. Jr.; Ren X.; Gottesfeld S.; Neutzler J. System issues for Nafion-based portable direct methanol fuel cells. Proceedings of the International Symposium on Direct Methanol Fuel Cells; Electrochemical Society Proceedings: Pennington, NJ. 2001; pp 221–230.

[ref73] Hickner M. A. Transport and structure in fuel cell proton exchange membranes. Ph.D. Thesis, Virginia Polytechnic Institute and State University, Blacksburg, VA, 2003.

[ref74] Kim Y. S.; Sumner M. J.; Harrison W. L.; Riffle J. S.; McGrath J. E.; Pivovar B. S. Direct Methanol Fuel Cell Performance of Disulfonated Poly(arylene ether benzonitrile) Copolymers. J. Electrochem. Soc. 2004, 151, A2150 10.1149/1.1819837.

[ref75] Weng D.; Wainright J. S.; Landau U.; Savinell R. F. Electro-osmotic Drag Coefficient of Water and Methanol in Polymer Electrolytes at Elevated Temperatures. J. Electrochem. Soc. 1996, 143, 1260–1263.

[ref76] Balko E.; McElroy J.; LaConti A. Halogen acid electrolysis in solid polymer electrolyte cells. Int. J. Hydrogen Energy 1981, 6, 577–587. 10.1016/0360-3199(81)90023-9.

[ref77] Motupally S.; Becker A. J.; Weidner J. W. Water Transport in Polymer Electrolyte Membrane Electrolyzers Used to Recycle Anhydrous HCl. J. Electrochem. Soc. 2002, 149, D63 10.1149/1.1464135.

[ref78] Trivijitkasem P.; Østvold T. Water transport in ion exchange membranes. Electrochim. Acta 1980, 25, 171–178. 10.1016/0013-4686(80)80039-9.

[ref79] Okada T.; Kjelstrup Ratkje S.; Hanche-Olsen H. Water transport in cation exchange membranes. J. Membr. Sci. 1992, 66, 179–192. 10.1016/0376-7388(92)87008-L.

[ref80] Fuller T. F.; Newman J. Experimental Determination of the Transport Number of Water in Nafion 117 Membrane. J. Electrochem. Soc. 1992, 139, 1332–1337. 10.1149/1.2069407.

[ref81] Bouwman P. Electrochemical Hydrogen Compression (EHC) solutions for hydrogen infrastructure. Fuel Cells Bulletin 2014, 2014, 12–16. 10.1016/S1464-2859(14)70149-X.

