# Supplementary material for: Electro-osmotic Drag and Thermodynamic Properties of Water in Hydrated Nafion Membranes from Molecular Dynamics
Source: J Phys Chem C Nanomater Interfaces. 2022 May 3;126(18):8121–33. doi: 10.1021/acs.jpcc.2c01226 (PMC9109139; doi:10.1021/acs.jpcc.2c01226)
Supplement: Supplementary file 1 — jp2c01226_si_001.pdf [file jp2c01226_si_001.pdf]

Electro-osmotic Drag and Thermodynamic Properties of Water in Hydrated Nafion Membranes from Molecular Dynamics

Ahmadreza Rahbari,[†,‡] Remco Hartkamp,[†] Othonas A. Moultos,[†] Albert Bos,[‡] Leo J. P. van den Broeke,[†] Mahinder Ramdin,[†] David Dubbeldam,[¶] Alexey V. Lyulin,[§,‖] and Thijs J. H. Vlugt[*,†]

†*Engineering Thermodynamics, Process & Energy Department, Faculty of Mechanical, Maritime and Materials Engineering, Delft University of Technology, Leeghwaterstraat 39, 2628CB, Delft, The Netherlands*

‡*XINTC global, Wesselseweg 134, 3774 RL Kootwijkerbroek, The Netherlands*

¶*Van't Hoff Institute for Molecular Sciences, University of Amsterdam, Science Park 904, 1098XH Amsterdam, The Netherlands*

§*Soft Matter and Biological Physics, Department of Applied Physics, Eindhoven University of Technology, P.O. Box 513, 5600 MB Eindhoven, The Netherlands*

‖*Center for Computational Energy Research, P.O. Box 6336, 5600 HH Eindhoven, The Netherlands*

E-mail: t.j.h.vlugt@tudelft.nl

Table S1: Ratio of the ensemble average velocity of water to the ensemble average velocity of hydronium, $\langle v_{H_2O} \rangle / \langle v_{H_3O^+} \rangle$, as a function of temperature and electric field strength for $\lambda = 5$. The electric field is imposed along the x, y or z axis in independent simulations. Two starting independent configurations 1 and 2 were used.

T/[K]	e/[V/Å]	Axis	Configuration	$\langle v_{H_2O} \rangle / \langle v_{H_3O^+} \rangle$
330	0.020	x	1	0.372
330	0.020	x	2	0.421
330	0.020	y	1	0.282
330	0.020	y	2	0.317
330	0.020	z	1	0.441
330	0.020	z	2	0.421
330	0.050	x	1	0.494
330	0.050	x	2	0.507
330	0.050	y	1	0.422
330	0.050	y	2	0.456
330	0.050	z	1	0.453
330	0.050	z	2	0.537
330	0.075	x	1	0.493
330	0.075	x	2	0.431
330	0.075	y	1	0.457
330	0.075	y	2	0.465
330	0.075	z	1	0.424
330	0.075	z	2	0.445
330	0.100	x	1	0.432
330	0.100	x	2	0.415
330	0.100	y	1	0.429
330	0.100	y	2	0.410
330	0.100	z	1	0.426
330	0.100	z	2	0.454
360	0.020	x	1	0.400
360	0.020	x	2	0.319
360	0.020	y	1	0.338
360	0.020	y	2	0.374
360	0.020	z	1	0.425
360	0.020	z	2	0.552
360	0.050	x	1	0.518
360	0.050	x	2	0.454
360	0.050	y	1	0.429
360	0.050	y	2	0.451
360	0.050	z	1	0.471

Continued on next page

$T/[\mathrm{K}]$	$e/[V/\mathrm{Å}]$	Axis	Configuration	$\langle v_{H_2O}\rangle / \langle v_{H_3O^+}\rangle$
360	0.050	z	2	0.508
360	0.075	x	1	0.486
360	0.075	x	2	0.445
360	0.075	y	1	0.473
360	0.075	y	2	0.487
360	0.075	z	1	0.459
360	0.075	z	2	0.456
360	0.100	x	1	0.433
360	0.100	x	2	0.415
360	0.100	y	1	0.429
360	0.100	y	2	0.434
360	0.100	z	1	0.432
360	0.100	z	2	0.409
420	0.020	x	1	0.421
420	0.020	x	2	0.376
420	0.020	y	1	0.349
420	0.020	y	2	0.348
420	0.020	z	1	0.380
420	0.020	z	2	0.417
420	0.050	x	1	0.436
420	0.050	x	2	0.434
420	0.050	y	1	0.393
420	0.050	y	2	0.411
420	0.050	z	1	0.418
420	0.050	z	2	0.425
420	0.075	x	1	0.427
420	0.075	x	2	0.413
420	0.075	y	1	0.438
420	0.075	y	2	0.412
420	0.075	z	1	0.419
420	0.075	z	2	0.416
420	0.100	x	1	0.403
420	0.100	x	2	0.402
420	0.100	y	1	0.398
420	0.100	y	2	0.405
420	0.100	z	1	0.415
420	0.100	z	2	0.399

Table S2: Ratio of the ensemble average velocity of water to the ensemble average velocity of hydronium, $\langle v_{H_2O} \rangle / \langle v_{H_3O^+} \rangle$, as a function of temperature and electric field strength for $\lambda = 10$. The electric field is imposed along the x, y or z axis in independent simulations. Two starting independent configurations 1 and 2 were used.

T/[K]	e/[V/Å]	Axis	Configuration	$\langle v_{H_2O} \rangle / \langle v_{H_3O^+} \rangle$
330	0.020	x	1	0.355
330	0.020	x	2	0.347
330	0.020	y	1	0.421
330	0.020	y	2	0.442
330	0.020	z	1	0.565
330	0.020	z	2	0.547
330	0.050	x	1	0.450
330	0.050	x	2	0.470
330	0.050	y	1	0.478
330	0.050	y	2	0.463
330	0.050	z	1	0.538
330	0.050	z	2	0.565
330	0.075	x	1	0.394
330	0.075	x	2	0.420
330	0.075	y	1	0.397
330	0.075	y	2	0.384
330	0.075	z	1	0.439
330	0.075	z	2	0.470
330	0.100	x	1	0.383
330	0.100	x	2	0.371
330	0.100	y	1	0.363
330	0.100	y	2	0.365
330	0.100	z	1	0.378
330	0.100	z	2	0.371
360	0.020	x	1	0.359
360	0.020	x	2	0.322
360	0.020	y	1	0.465
360	0.020	y	2	0.434
360	0.020	z	1	0.470
360	0.020	z	2	0.523
360	0.050	x	1	0.463
360	0.050	x	2	0.407
360	0.050	y	1	0.437
360	0.050	y	2	0.469
360	0.050	z	1	0.434

Continued on next page

$T/[\mathrm{K}]$	$e/[V/\text{Å}]$	Axis	Configuration	$\langle v_{\mathrm{H_2O}} \rangle / \langle v_{\mathrm{H_3O^+}} \rangle$
360	0.050	z	2	0.475
360	0.075	x	1	0.402
360	0.075	x	2	0.389
360	0.075	y	1	0.395
360	0.075	y	2	0.397
360	0.075	z	1	0.428
360	0.075	z	2	0.419
360	0.100	x	1	0.375
360	0.100	x	2	0.371
360	0.100	y	1	0.359
360	0.100	y	2	0.367
360	0.100	z	1	0.375
360	0.100	z	2	0.371
420	0.020	x	1	0.392
420	0.020	x	2	0.394
420	0.020	y	1	0.421
420	0.020	y	2	0.400
420	0.020	z	1	0.406
420	0.020	z	2	0.469
420	0.050	x	1	0.432
420	0.050	x	2	0.410
420	0.050	y	1	0.424
420	0.050	y	2	0.402
420	0.050	z	1	0.421
420	0.050	z	2	0.444
420	0.075	x	1	0.386
420	0.075	x	2	0.394
420	0.075	y	1	0.402
420	0.075	y	2	0.399
420	0.075	z	1	0.384
420	0.075	z	2	0.379
420	0.100	x	1	0.367
420	0.100	x	2	0.360
420	0.100	y	1	0.359
420	0.100	y	2	0.361
420	0.100	z	1	0.374
420	0.100	z	2	0.361

Table S3: Ratio of the ensemble average velocity of water to the ensemble average velocity of hydronium, $\langle v_{H_2O} \rangle / \langle v_{H_3O^+} \rangle$, as a function of temperature and electric field strength for $\lambda = 15$. The electric field is imposed along the x, y or z axis in independent simulations. Two starting independent configurations 1 and 2 were used.

$T/[K]$	$e/[V/Å]$	Axis	Configuration	$\langle v_{H_2O} \rangle / \langle v_{H_3O^+} \rangle$
330	0.020	x	1	0.400
330	0.020	x	2	0.379
330	0.020	y	1	0.399
330	0.020	y	2	0.405
330	0.020	z	1	0.385
330	0.020	z	2	0.353
330	0.050	x	1	0.399
330	0.050	x	2	0.394
330	0.050	y	1	0.428
330	0.050	y	2	0.430
330	0.050	z	1	0.397
330	0.050	z	2	0.416
330	0.075	x	1	0.388
330	0.075	x	2	0.410
330	0.075	y	1	0.374
330	0.075	y	2	0.391
330	0.075	z	1	0.387
330	0.075	z	2	0.395
330	0.100	x	1	0.351
330	0.100	x	2	0.351
330	0.100	y	1	0.362
330	0.100	y	2	0.341
330	0.100	z	1	0.330
330	0.100	z	2	0.355
360	0.020	x	1	0.384
360	0.020	x	2	0.360
360	0.020	y	1	0.409
360	0.020	y	2	0.421
360	0.020	z	1	0.365
360	0.020	z	2	0.365
360	0.050	x	1	0.409
360	0.050	x	2	0.376
360	0.050	y	1	0.443
360	0.050	y	2	0.401
360	0.050	z	1	0.389

Continued on next page

$T/[\mathrm{K}]$	$e/[V/\mathrm{Å}]$	Axis	Configuration	$\langle v_{\mathrm{H_2O}} \rangle / \langle v_{\mathrm{H_3O^+}} \rangle$
360	0.050	z	2	0.397
360	0.075	x	1	0.389
360	0.075	x	2	0.415
360	0.075	y	1	0.367
360	0.075	y	2	0.405
360	0.075	z	1	0.405
360	0.075	z	2	0.430
360	0.100	x	1	0.343
360	0.100	x	2	0.357
360	0.100	y	1	0.348
360	0.100	y	2	0.355
360	0.100	z	1	0.345
360	0.100	z	2	0.350
420	0.020	x	1	0.394
420	0.020	x	2	0.397
420	0.020	y	1	0.400
420	0.020	y	2	0.414
420	0.020	z	1	0.383
420	0.020	z	2	0.395
420	0.050	x	1	0.407
420	0.050	x	2	0.419
420	0.050	y	1	0.429
420	0.050	y	2	0.496
420	0.050	z	1	0.445
420	0.050	z	2	0.403
420	0.075	x	1	0.386
420	0.075	x	2	0.386
420	0.075	y	1	0.387
420	0.075	y	2	0.387
420	0.075	z	1	0.392
420	0.075	z	2	0.379
420	0.100	x	1	0.343
420	0.100	x	2	0.341
420	0.100	y	1	0.345
420	0.100	y	2	0.340
420	0.100	z	1	0.341
420	0.100	z	2	0.344

Table S4: Ratio of the ensemble average velocity of water to the ensemble average velocity of hydronium, $\langle v_{H_2O} \rangle / \langle v_{H_3O^+} \rangle$, as a function of temperature and electric field strength for $\lambda = 20$. The electric field is imposed along the x, y or z axis in independent simulations. Two starting independent configurations 1 and 2 were used.

T/[K]	e/[V/Å]	Axis	Configuration	$\langle v_{H_2O} \rangle / \langle v_{H_3O^+} \rangle$
330	0.020	x	1	0.450
330	0.020	x	2	0.463
330	0.020	y	1	0.348
330	0.020	y	2	0.354
330	0.020	z	1	0.360
330	0.020	z	2	0.357
330	0.050	x	1	0.445
330	0.050	x	2	0.394
330	0.050	y	1	0.377
330	0.050	y	2	0.411
330	0.050	z	1	0.387
330	0.050	z	2	0.446
330	0.075	x	1	0.383
330	0.075	x	2	0.364
330	0.075	y	1	0.357
330	0.075	y	2	0.382
330	0.075	z	1	0.393
330	0.075	z	2	0.386
330	0.100	x	1	0.328
330	0.100	x	2	0.329
330	0.100	y	1	0.325
330	0.100	y	2	0.330
330	0.100	z	1	0.323
330	0.100	z	2	0.326
360	0.020	x	1	0.428
360	0.020	x	2	0.448
360	0.020	y	1	0.363
360	0.020	y	2	0.364
360	0.020	z	1	0.355
360	0.020	z	2	0.341
360	0.050	x	1	0.455
360	0.050	x	2	0.394
360	0.050	y	1	0.533
360	0.050	y	2	0.406
360	0.050	z	1	0.431

Continued on next page

$T/[\mathrm{K}]$	$e/[V/\mathrm{\AA}]$	Axis	Configuration	$\langle v_{\mathrm{H_2O}} \rangle / \langle v_{\mathrm{H_3O^+}} \rangle$
360	0.050	z	2	0.408
360	0.075	x	1	0.382
360	0.075	x	2	0.370
360	0.075	y	1	0.399
360	0.075	y	2	0.394
360	0.075	z	1	0.393
360	0.075	z	2	0.372
360	0.100	x	1	0.338
360	0.100	x	2	0.338
360	0.100	y	1	0.336
360	0.100	y	2	0.335
360	0.100	z	1	0.339
360	0.100	z	2	0.329
420	0.020	x	1	0.428
420	0.020	x	2	0.431
420	0.020	y	1	0.356
420	0.020	y	2	0.365
420	0.020	z	1	0.376
420	0.020	z	2	0.365
420	0.050	x	1	0.412
420	0.050	x	2	0.399
420	0.050	y	1	0.499
420	0.050	y	2	0.388
420	0.050	z	1	0.389
420	0.050	z	2	0.402
420	0.075	x	1	0.379
420	0.075	x	2	0.380
420	0.075	y	1	0.363
420	0.075	y	2	0.362
420	0.075	z	1	0.376
420	0.075	z	2	0.362
420	0.100	x	1	0.327
420	0.100	x	2	0.328
420	0.100	y	1	0.330
420	0.100	y	2	0.328
420	0.100	z	1	0.327
420	0.100	z	2	0.329

Table S5: Ratio between the average velocities of water molecules and hydronium ions, and the electro-osmotic drag coefficient (ξ_D) as a function of water content, λ, and temperature. σ is the standard deviation of the mean from 6 independent simulations.

λ	T/[K]	$\langle v_{H_2O} \rangle / \langle v_{H_3O^+} \rangle$	σ	ξ_D	σ
5	330	0.43	0.04	2.2	0.2
5	360	0.44	0.03	2.2	0.2
5	420	0.41	0.02	2.0	0.1
10	330	0.4	0.1	4	1
10	360	0.41	0.03	4.1	0.3
10	420	0.40	0.03	4.0	0.3
15	330	0.38	0.03	5.8	0.4
15	360	0.38	0.02	5.8	0.4
15	420	0.39	0.04	5.8	0.6
20	330	0.38	0.04	8	1
20	360	0.39	0.04	7.7	0.8
20	420	0.38	0.04	7.5	0.7

Table S6: Computed partial molar volumes and and partial molar excess enthalpies of water as a function of water uptake in the Nafion membrane. Uncertainties (σ) are calculated using block averaging (5 blocks). ($\bar{h}^{\mathrm{ig}}_{\mathrm{H_2O}}$) is the ideal gas reference enthalpy of water which can be found in JANAF tables[1,2] or REFPROP.[3] The molar properties of pure water computed using the CFF force field are (in units of cm^3/mol and kJ/mol, respectively) $\overline{v}_{\mathrm{H_2O}} = 19.76(2)$, $\overline{h}_{\mathrm{H_2O}} = -28.29(1)$ at $T = 330$ K, $\overline{v}_{\mathrm{H_2O}} = 20.45(1)$, $\overline{h}_{\mathrm{H_2O}} = -26.57(1)$ at $T = 360$ K. At $T = 420$ K, the saturation pressure for the liquid phase is higher than 1 atm, and the molar properties of pure liquid water at $T = 420$ K are not considered here.

λ	T/[K]	$\bar{v}_{\mathrm{H_2O}}$/[cm^3/mol]	$\sigma_{\bar{v}}$	$(\bar{h}_{\mathrm{H_2O}} - \bar{h}^{\mathrm{ig}}_{\mathrm{H_2O}})$/[kJ/mol]	$\sigma_{\bar{h}}$
5	330	16.3	0.8	-35.6	0.7
10	330	17.5	0.8	-34.7	0.4
15	330	18.2	1.0	-34.7	0.6
20	330	18.5	0.3	-34.7	0.5
5	360	16	1	-34.3	0.6
10	360	18.9	0.4	-32.2	0.6
15	360	18.1	0.9	-32.7	0.6
20	360	19.8	0.7	-31.4	0.5
5	420	18	2	-27.2	1.0
10	420	21.1	0.8	-27	1
15	420	21	1	-29	1
20	420	19.9	0.4	-28.5	0.5
5	570	25.4	0.7	-18	1
10	570	25	2	-18	1
15	570	29	2	-16	2
20	570	31	3	-15	3

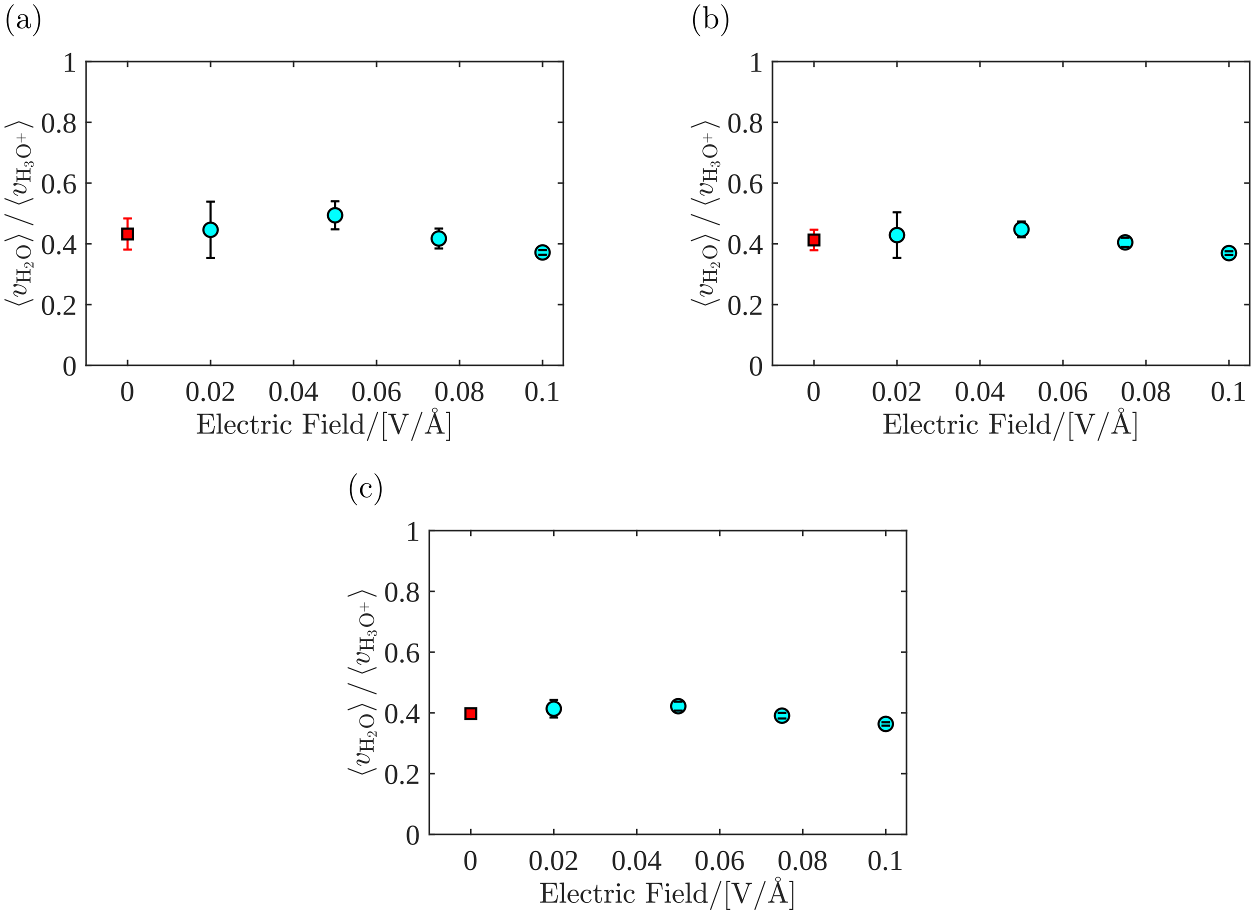

Figure S1: Ratio between the velocities of water and hydronium for $\lambda = 10$ at different magnitudes of the electric field imposed on the simulation box at (a) $T = 330$ K, (b) $T = 360$ K, and (c) $T = 420$ K. Squares are the average over the ratio between the velocities. Raw data are provided in Table S2.

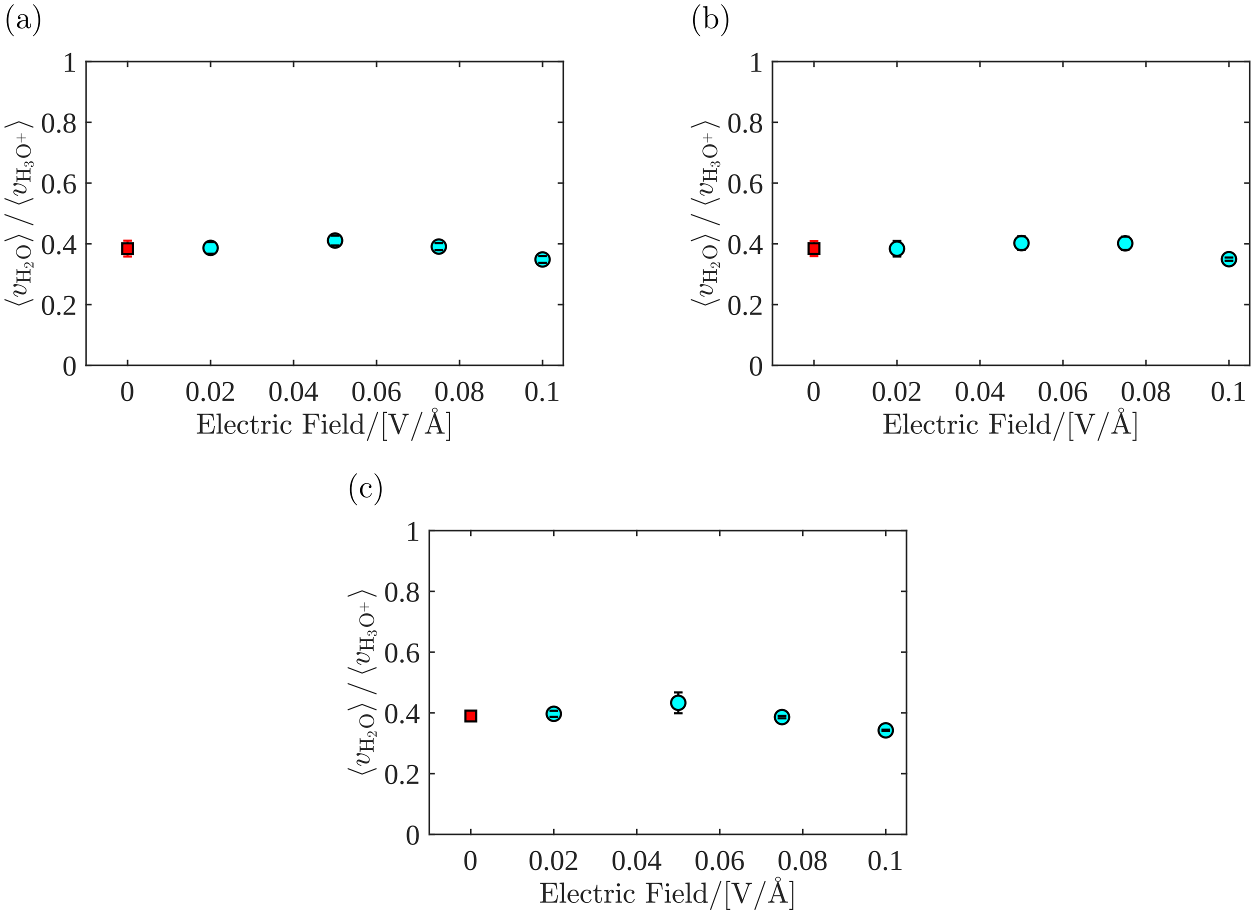

Figure S2: Ratio between the velocities of water and hydronium for $\lambda = 15$ at different magnitudes of the electric field imposed on the simulation box at (a) $T = 330$ K, (b) $T = 360$ K, and (c) $T = 420$ K. Squares are the average over the ratio between the velocities. Raw data are provided in Table S3.

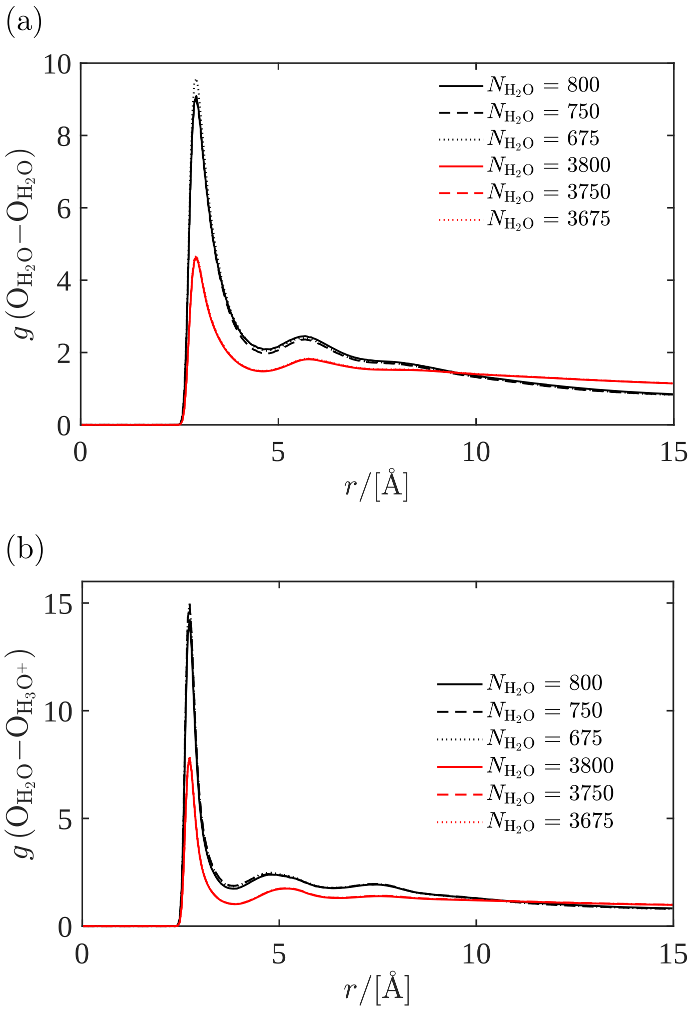

Figure S3: Radial distribution functions for water and hydronium in hydrated Nafion computed for different number of water molecules at $T = 330$ K. Different configurations were generated by removing water molecules from the simulation box equilibrated at $\lambda = 5$ (800 water molecules), and $\lambda = 20$ (3800 water molecules)[4] : (a) water-water (b) water-hydrionium.

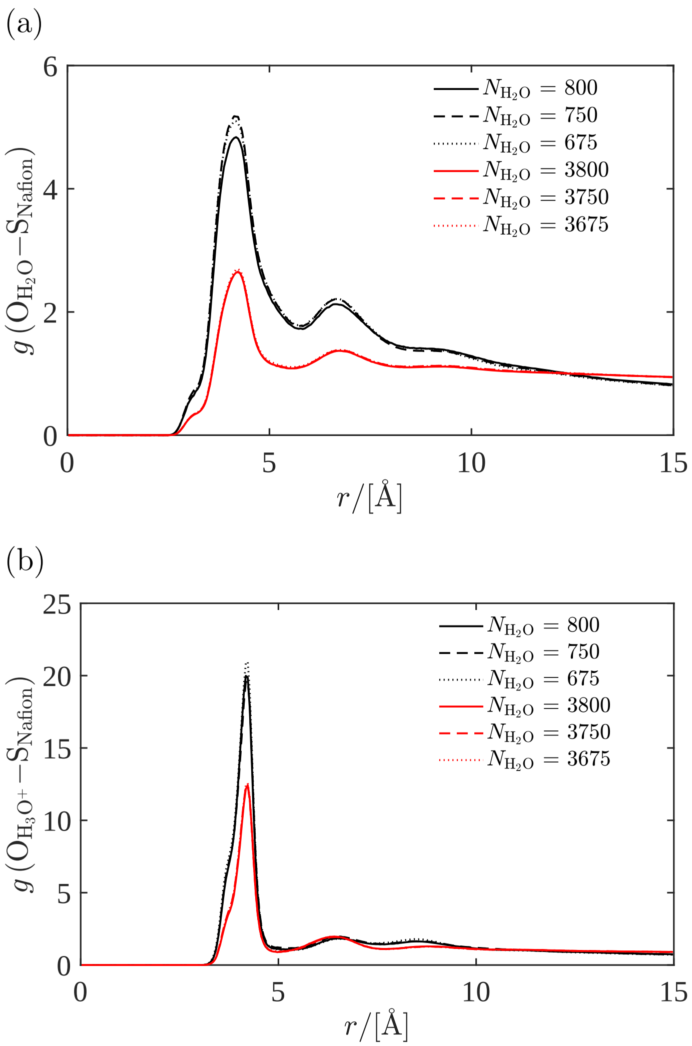

Figure S4: Radial distribution functions for water and hydronium in hydrated Nafion computed for different number of water molecules at $T = 330$ K. Different configurations were generated by removing water molecules from the simulation box equilibrated at $\lambda = 5$ (800 water molecules), and $\lambda = 20$ (3800 water molecules)[4] : (a) water-Sulfur (b) hydronium-Sulfur.

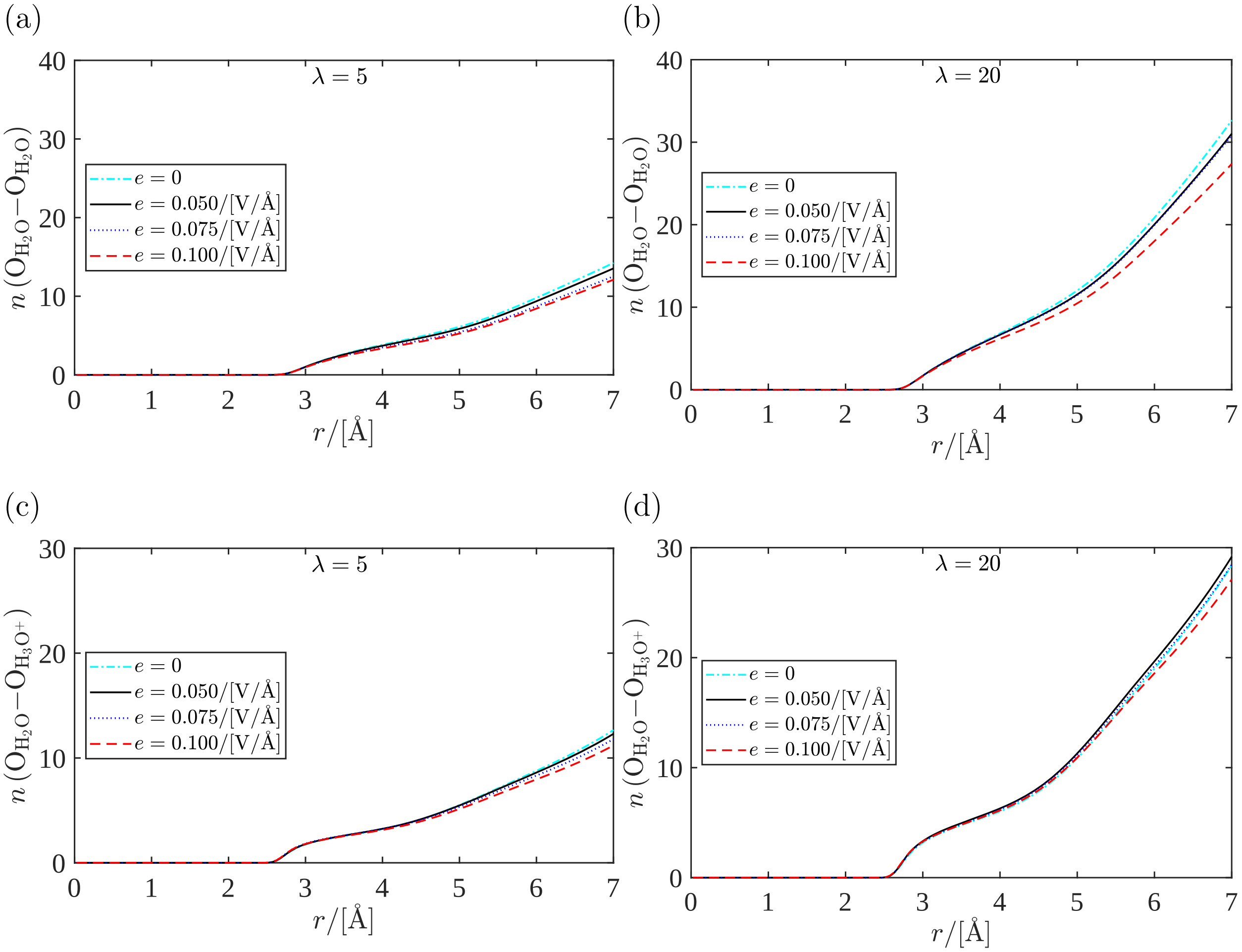

Figure S5: Coordination numbers for different λ corresponding to Fig. 5 of the main text. The coordination number $n(i-j)$ of species j around species i is defined as: $n(i-j) = 4\pi \int_0^r \mathrm{d}r\, r^2 \rho_j g_{ij}(r)$ in which ρ_j is the number density of species j.

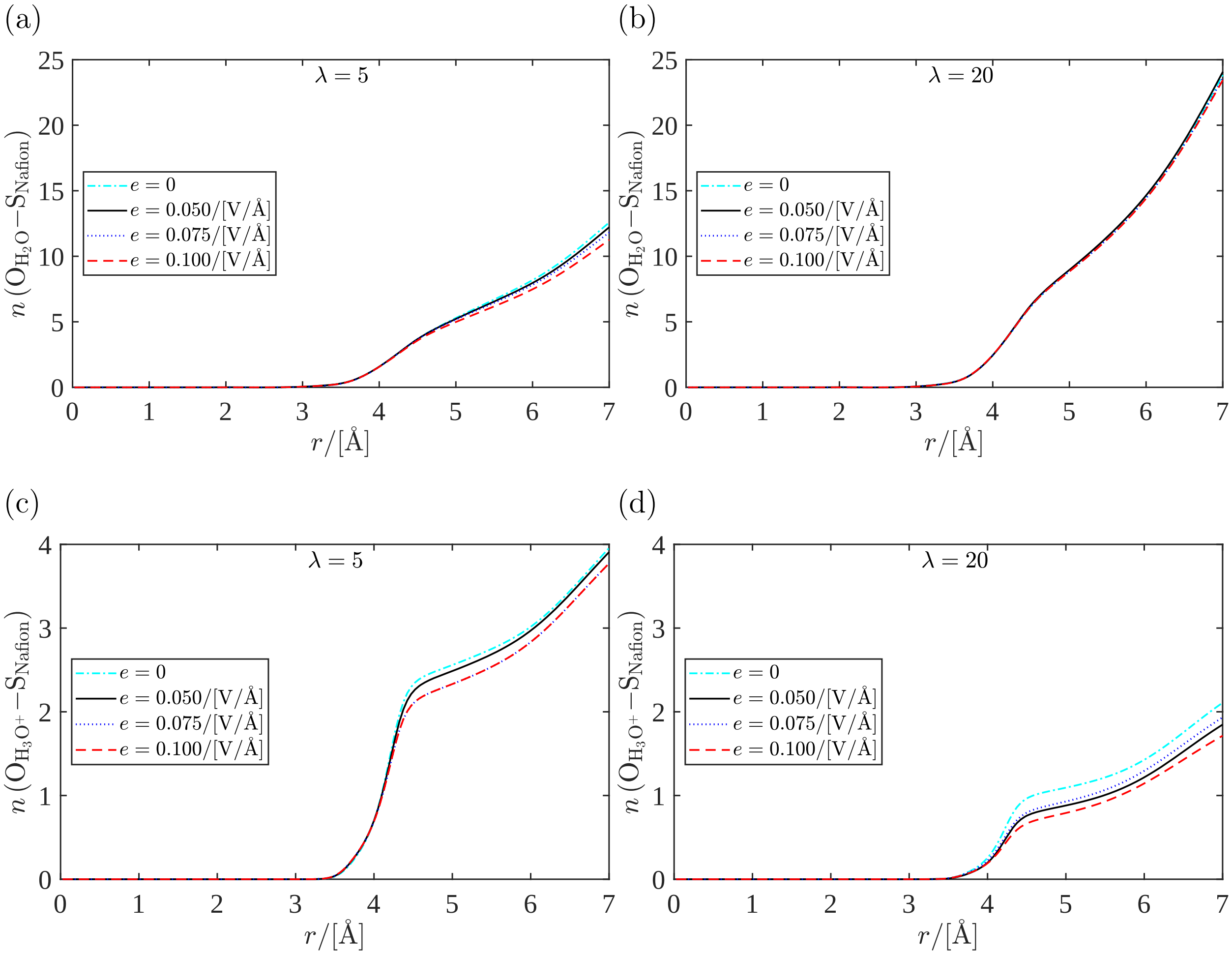

Figure S6: Coordination numbers for different λ corresponding to Fig. 6 of the main text. The coordination number $n(i-j)$ of species j around species i is defined as: $n(i-j) = 4\pi \int_0^r \mathrm{d}r\, r^2 \rho_j g_{ij}(r)$ in which ρ_j is the number density of species j.

References

(1) Chase, M. W. NIST-JANAF Themochemical Tables, Fourth Edition. *J. Phys. Chem. Ref. Data* **1998**, *4*, 1–1951.

(2) Chase, M. W.; Curnutt, J.; Prophet, H.; McDonald, R.; Syverud, A. JANAF thermochemical tables, 1975 supplement. *J. Phys. Chem. Ref. Data.* **1975**, *4*, 1–176.

(3) Lemmon, E. W.; Huber, M. L.; McLinden, M. O. NIST reference fluid thermodynamic and transport properties–REFPROP. *NIST standard reference database* **2002**, *23*, v7, `https://www.nist.gov/srd/refprop`. Accessed: 07-01-2021.

(4) Lyulin, A. V.; Sengupta, S.; Varughese, A.; Komarov, P.; Venkatnathan, A. Effect of Annealing on Structure and Diffusion in Hydrated Nafion Membranes. *ACS Appl. Polym. Mater.* **2020**, *2*, 5058–5066.
